# Lung Cancer Screening in a Population from Northeast Italy Exposed to Both Asbestos and Smoking: A Cost-Effectiveness Analysis

**DOI:** 10.3390/jcm15083136

**Published:** 2026-04-20

**Authors:** Rami Cosulich, Chloe Thomas, Fabiano Barbiero, Duncan Gillespie, Ettore Bidoli, Maria Assunta Cova, Stefano Lovadina, Alessandra Guglielmi, Luigino Dal Maso, Barbara Alessandrini, Francesca Larese Filon, Fabio Barbone, Elisa Baratella

**Affiliations:** 1 Department of Statistical Sciences, University of Padua, 35121 Padova, Italy; 2 Sheffield Centre for Health and Related Research (SCHARR), School of Medicine and Population Health, The University of Sheffield, Sheffield S1 4DA, UK; c.thomas@sheffield.ac.uk (C.T.); duncan.gillespie@sheffield.ac.uk (D.G.); 3 Unit of Occupational Medicine, University of Trieste, 34129 Trieste, Italy; fabiano.barbiero@units.it (F.B.); larese@units.it (F.L.F.); 4 Cancer Epidemiology Unit, Centro di Riferimento Oncologico di Aviano (CRO) IRCCS, 33081 Aviano, Italy; bidolie@cro.it (E.B.); dalmaso@cro.it (L.D.M.); 5 Radiology Unit, Department of Medicine, Surgery and Health Sciences, University Hospital of Cattinara, 34149 Trieste, Italy; m.cova@fmc.units.it (M.A.C.); elisa.baratella@units.it (E.B.); 6 Thoracic Surgery Unit, Azienda Sanitaria Universitaria Giuliano Isontina, University Hospital of Cattinara, 34149 Trieste, Italy; stefano.lovadina@asugi.sanita.fvg.it; 7 Oncology Unit, 34134 Trieste, Italy; alessandra.guglielmi@asugi.sanita.fvg.it; 8 Struttura Operativa Complessa Prevenzione e Sicurezza nei Luoghi di Lavoro, Azienda Sanitaria Universitaria Friuli Centrale, 33100 Udine, Italy; barbara.alessandrini@regione.fvg.it; 9 Centro Interdipartimentale di Scienze Mediche, Azienda Sanitaria Universitaria Integrata del Trentino, University of Trento, 38100 Trento, Italy; fabio.barbone@unitn.it

**Keywords:** lung cancer, screening, asbestos, cost-effectiveness, cost, QALY, decision tree, Markov model, economic model, overdiagnosis

## Abstract

**Background**: Past workplace exposure to asbestos in combination with tobacco smoking has increased the risk of lung cancer for some residents in an area within the Friuli Venezia Giulia region, Northeast Italy. In light of studies showing that lung cancer screening (LCS) with low-dose computed tomography (LDCT) can reduce mortality, local stakeholders and decision-makers decided to assess the potential benefits, harms and cost-effectiveness of a single round of LCS with LDCT versus standard care among people aged 55 to 80 who were formerly exposed to asbestos and with at least 10 pack-years of smoking. **Methods**: An economic model was developed using a decision tree connected to a Markov cohort model. The primary outcome was the incremental cost per additional quality-adjusted life year (QALY). Other outcomes included the number of life years saved, the number of deaths averted and overdiagnosis. **Results**: Per 10,000 people screened, the intervention led to 395 additional QALYs (95% credible interval: 129 to 831) and incremental total costs of EUR 1,086,345 (95% credible interval: −852,607 to 2,155,826). The incremental cost per QALY gained was EUR 2750. There was a probability of cost-effectiveness of 99.5% relative to a threshold of EUR 25,000. **Conclusions**: The model estimated that the intervention was cost-effective. The model’s simplifications and limitations should be considered when interpreting the findings in relation to policy-making decisions. Further research could include the costs and benefits of incidental findings and could assess the cost-effectiveness of repeated rounds of screening for the same population.

## 1. Introduction

Exposure to asbestos fibres in the air is a known cause of lung cancer [1], as is tobacco smoking [2], and exposure to both asbestos and smoking has a combined effect on lung cancer that is greater than the sum of the individual effects [3]. Trends in asbestos supply and consumption have seen a rapid decrease since 1985 [4], and asbestos was banned in Italy in 1992 [5]. Prior to 1985, many workers were exposed to it in the areas of Trieste and Monfalcone within the Friuli Venezia Giulia (FVG) region, Northeast Italy: Monfalcone was a major industrial centre including a large shipyard, while in Trieste, the regional capital, there was an industrial complex producing cast iron. Since 2020, both areas have been under the responsibility of a health authority called Azienda Sanitaria Universitaria Giuliano Isontina (ASUGI). Considering that asbestos can cause lung cancer many years after the time of exposure [6], in 2023 ASUGI decided to start an assessment of the potential benefits, harms and cost-effectiveness of lung cancer screening (LCS) among people currently or formerly smoking and who were formerly exposed to asbestos.

As reported in a recent review on LCS [7], randomised controlled trials (RCTs) have shown that screening with low-dose computed tomography (LDCT) can reduce lung cancer-related mortality, compared to chest radiography screening or no screening. Most RCTs did not consider asbestos among the selection criteria, although there were some exceptions: a trial in China included occupational exposure to carcinogenic agents as a selection criterion [8]; the United Kingdom Lung Screen (UKLS) trial used Version 2 of the Liverpool Lung Project (LLPv2) risk score, which takes into account occupational exposure to asbestos [9,10,11]. The trial in China found an increase in the detection of early-stage lung cancer with screening during a two-year follow-up [8]. In the UKLS trial, cumulative mortality from lung cancer was lower in the screening arm [9]. In addition, one observational study on men exposed to asbestos in the ASUGI area found a strong reduction in lung cancer mortality with LDCT screening [12].

A review found that the cost-effectiveness estimates of LCS varied considerably between different studies and settings [7]. Among other factors, cost-effectiveness was linked to the participant selection criteria and the burden of lung cancer among the eligible population [7]. To this date, to our knowledge, only two studies have evaluated the cost-effectiveness of LCS in the Italian context [13,14]. Both studies focused on people with at least 30 pack-years, who either currently smoked or had stopped smoking within the previous 15 years. Neither of the two studies mentioned asbestos exposure. Beyond the Italian context, based on a review of health economic evaluations of chest LDCT screening [15], only one previous study, in Canada, has focused on the cost-effectiveness of LDCT LCS among asbestos-exposed individuals [16]. The study found that biennial LDCT screening was cost-effective for people aged 55–74 who fulfilled both of these criteria: (1) an asbestos exposure leading to a relative risk of 2 compared to the non-exposed in relation to the lung cancer outcome; (2) at least 15 pack-years of smoking.

Two reviews focused on the eligibility criteria that should be applied to people formerly exposed to asbestos for participation in an LCS programme [17,18]. Both reviews concluded that screening should be aimed at people who have at least one other risk factor in addition to asbestos exposure [17,18]: one review highlighted having ever smoked as an additional risk factor [17], while the other review recommended a minimum threshold of 10 pack-years of smoking or other risk factors such as asbestos-related fibrosis or chronic lung disease, in addition to a minimum duration or level of asbestos exposure [18].

Considering the importance of the combined effect of asbestos and smoking on lung cancer [3], and a lack of cost-effectiveness studies evaluating LCS specifically among people exposed to asbestos outside Canada, the current work aimed to assess the potential benefits, harms and cost-effectiveness of LCS among people living in the ASUGI area with both former exposure to asbestos and at least 10-pack years of smoking. Moreover, this study aimed to evaluate the cost-effectiveness of LCS in different sex and age subgroups.

## 2. Materials and Methods

A new health economic model was developed through a collaboration between health economists and the ASUGI LCS working group, composed of clinical and epidemiology experts.

### 2.1. Model Population, Intervention and Comparator

The model population was a cohort aged 55–80 formerly exposed to asbestos and currently or formerly smoking with at least 10 pack-years, living in the ASUGI area. The lower age limit was selected considering that exposure to asbestos had mostly occurred prior to 1985, while the upper limit was taken from published recommendations [19,20]. The model population was stratified into sex and age groups (55–59, 60–64, 65–69, 70–74, 75–79, 80) using proportions from the registry of people formerly exposed to asbestos in the FVG region [21]. Each subgroup aged through the model years.

The intervention in the model was a one-off LCS with LDCT. The comparator was standard care, which was summarised with the term “passive surveillance”, where people formerly exposed to asbestos received an initial personalised recommendation to attend for a check-up approximately every two or three years, but there was no reminder to come back. When people attended for a check-up, they had respiratory function tests and an X-ray, even if they had no symptoms. In the intervention arm of the model, passive surveillance was removed for one year following screening.

### 2.2. Model Structure

**Figure 1:**
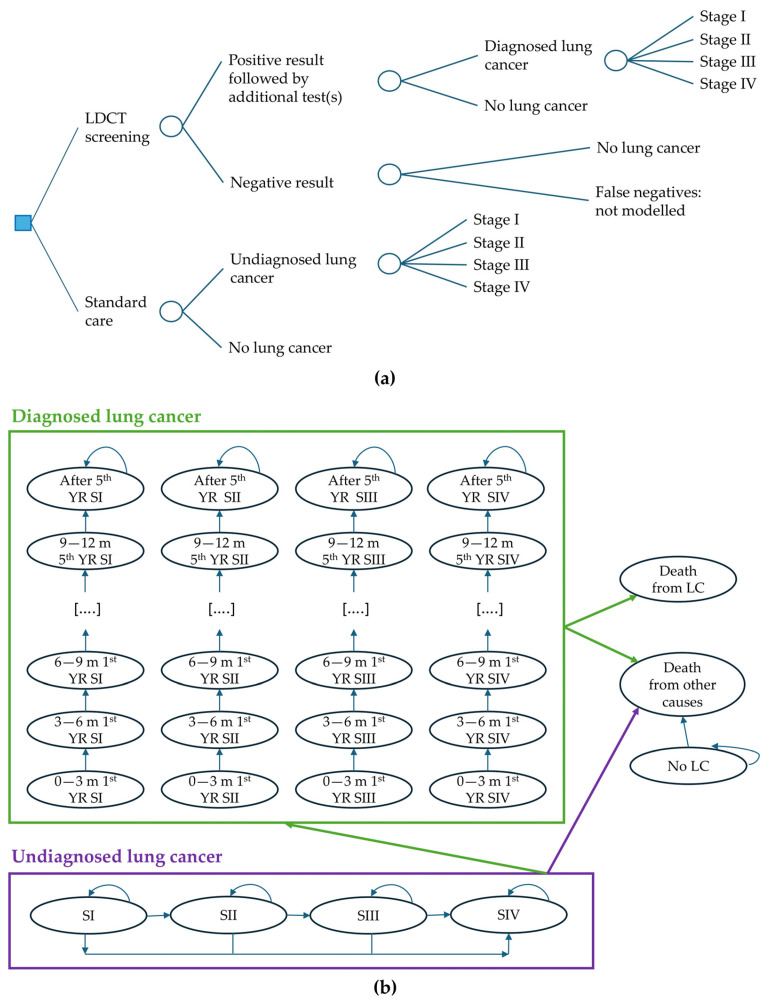
Model structure diagrams. Abbreviations: LC: lung cancer; LDCT: low-dose computed tomography; m: month; SI: stage I; SII: stage II; SIII: stage III; SIV: stage IV; YR: year. (**a**) Decision tree. A positive result includes probably benign, suspicious and highly suspicious results. Any positive results followed by additional tests that reveal an absence of lung cancer are called “false positives” in the current paper. (**b**) Markov model. Each circle is a different “health state”. People spend 3 months in each health state, with these exceptions: (1) people can stay for more than 3 months in states where there is an arrow that returns to the same circle; (2) death states (so-called “absorbing states”). The light green outline indicates diagnosed lung cancer. The purple outline indicates undiagnosed lung cancer.

The structure connected a decision tree (short-term model) and a Markov cohort model (long-term model), see Figure 1. The long-term model had a lifetime horizon, meaning that people were followed up until their death. The decision tree was used to determine how people were distributed across different health states at baseline. This distribution was used at the start of the Markov model, which used 3-month cycles. People with an initial false positive in the short-term model were simply considered as people with no lung cancer in the long-term model.

In the decision tree, the proportion of people with undiagnosed lung cancer (LC) in the standard care arm was equal to the proportion diagnosed with LC through screening in the intervention arm (screen-detectable prevalence). False negatives at screening were not modelled. This was a model simplification that did not affect the incremental results because the same set of long-term outcomes was expected for false negative results in the intervention arm as for lung cancers in the standard care arm, and the proportion of lung cancers in the standard care arm was implicitly reduced by an amount equivalent to the false negatives excluded from the intervention arm; see Supplementary File S2 for details. The stage distribution of undiagnosed LC in the standard care arm at the start of the model was equal to the stage distribution of diagnosed LC in the screening arm.

The whole Markov model in Figure 1 applied to the standard care arm, while for the intervention arm, undiagnosed lung cancer was not included—see Figure S1 in Supplementary File S2 for details. In both the intervention and standard care arms, after people were diagnosed in a specific stage, they were assumed to remain in that same diagnosed stage until death: relapse and remission were not explicitly modelled, but the probabilities of death from LC incorporated a proportion relapsing and a proportion being cured. From any of the diagnosed cancer stages, people could transition to death from LC or to death from other causes. From any of the undiagnosed stages, people could transition to diagnosis or to death from other causes. From undiagnosed stage I, II and III, they could also progress to a higher stage. Because of this, more LCs in the standard care arm were diagnosed at later stages compared to the intervention arm. This is key in the model because different stages are characterised by different utilities and LC mortality, and different diagnostic and treatment costs, as described in Section 2.3.

### 2.3. Model Parameters

#### 2.3.1. Proportions and Transition Probabilities

Screen-detectable prevalence was calculated using an LDCT-based LCS study conducted within the ASUGI area on a mostly male population formerly exposed to asbestos [22]. Due to a lack of data on how screen-detectable prevalence may differ between men and women formerly exposed to asbestos with at least 10 pack-years, the model assumed that it was the same for men and women. See Supplementary File S3 for details regarding this assumption. In the absence of data on variation by age in screen-detectable prevalence, in the model this mirrored variation by age in incidence in the general population of the Trieste province; see Supplementary File S3 for details. Screen-detectable prevalence increased from age 55–59 to age 75–79, but it was lower among people aged 80 than among those aged 70–79. See Table S1 in Supplementary File S1 for details. Overall, average screen-detectable prevalence in the model population aged 55–80 was 0.0253.

It was assumed that, in the absence of screening, the stage distribution among people exposed to asbestos would be the same as in the general population, and if screening was implemented, the stage distribution would shift to earlier stages in the same way as for the general population. The stage distribution at diagnosis in the standard care arm was taken from an Italian study on 2050 cancers [23]. The stage distribution for the screening arm was taken from the one-off screening group in the UKLS trial [9]. See Table S1 in Supplementary File S1, and Supplementary File S4, for details.

The 3-month probability of progressing from undiagnosed stage I, II or III to higher undiagnosed stages and the probability of diagnosis from undiagnosed stage IV were taken from a previous model [24]. For stages I, II and III, the 3-month probability of diagnosis was calculated with iterative calculation. See Table S1 in Supplementary File S1, and Supplementary File S5, for details.

For the first 5 years since diagnosis, the probability of death from lung cancer depended on stage at diagnosis, years since diagnosis, sex, and age. The probabilities were calculated based on people diagnosed in the Trieste province in 2010–2019, using data from the regional cancer registry [25]. Due to limited sample size in the registry data, probabilities were calculated using wider age bands: 55–69 and 70–84. This data did not include stage at diagnosis, so the probabilities were adjusted by stage using an Italian study on 2050 cancers diagnosed between 2002 and 2005 [23]. See Table S2 in Supplementary File S1, and Supplementary Files S6 and S7, for details. From the sixth year since diagnosis, due to a model simplification, the 3-month probability of death from lung cancer was assumed to be the same across all stages, without adjustments for sex or age, for all model years until death. The probability was calculated from an Italian source on deaths from lung cancer between the sixth and tenth year since diagnosis [26]; see Table S2 and Supplementary File S7 for details.

The probability of death from causes other than lung cancer was adjusted for asbestos exposure and depended on age and sex. See Supplementary File S8 for details. It was assumed that anyone arriving to age 100 died at that age.

#### 2.3.2. Utilities

For people without lung cancer, due to a lack of data on utilities for people with asbestos exposure, utilities relating to the Italian general population aged 55 and over [27] were adjusted for smoking using a study from England [28]. Utilities for people with and without lung cancer were lower for women than for men, as per [27]. Moreover, utilities were adjusted by age using English data, given the lack of equivalent Italian data. More specifically, we used a study on people currently or formerly smoking in England, which showed lower utilities in the older age groups [29]. Based on this study, we used these cut-offs for utility adjustment: 55–64, 65–74 and 75+. The utility for lung cancer from a study conducted in Italy [30] was combined with ratios comparing the utilities of stage II, III and IV to stage I from a study conducted in the United States [31]. It was assumed that, following diagnosis at a given stage, the utility for that stage applied until death (with the relevant age declines). Disutilities linked to anxiety from participation in screening and to false positive results were taken from a previous UK model on LCS [32]. See Tables S3 and S4 in Supplementary File S1 for the utility parameters; see Supplementary File S9 for the calculation of the utilities.

#### 2.3.3. Costs

Costs were in euros (EUR) in 2023 prices. The unit costs were taken from documents available on the FVG regional authority website [33,34]. The Harmonised Index of Consumer Prices (HICP) for Italy was used for inflation, more specifically, one version for hospital services [35] and another one for outpatient services [36]; see Table S5 in Supplementary File S1 for details. Inflated costs are reported in Tables S6–S8 in Supplementary File S1, and costs before inflation are reported in Supplementary File S12.

**Figure 2:**
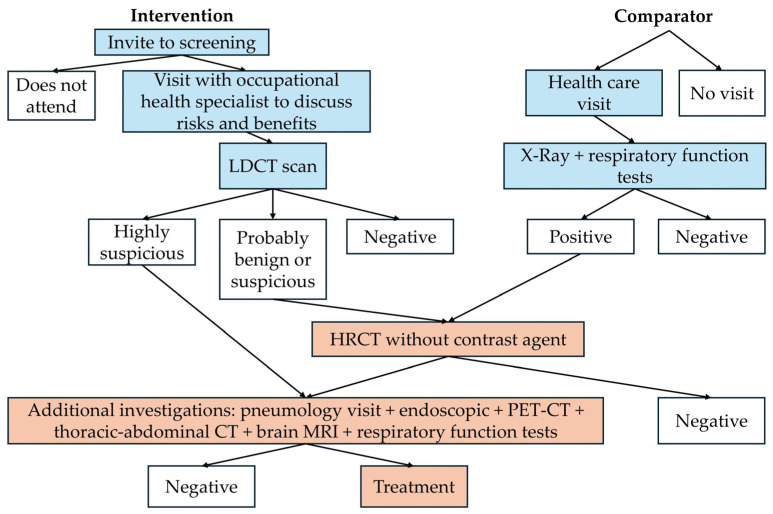
Simplified diagnostic pathways used in the model. Abbreviations: CT: computed tomography; HRCT: high resolution computed tomography; MRI: magnetic resonance imaging; PET: positron emission tomography. Colour legend: light blue colour: detection costs. Light pink colour: costs included in the calculation of the false-positive costs. The figure was drawn using a document describing local integrated diagnostic-treatment pathways [41] and with the involvement of local clinicians. See Supplementary File S10 for details on some simplifications made in the figure. LDCT results were classified using the Lung-RADS version 1.1 categorisation [42], as recommended in [19].

The blue boxes in Figure 2 show the detection costs, which, in the intervention arm, correspond to the upfront costs of screening. The only detection costs not included in the figure are the operating and administrative costs of screening, which were taken from a previous cost-effectiveness study of LCS among Italian smokers [13]. Overall, the detection cost per person in the intervention arm, i.e., per screening participant, was EUR 179. The detection cost per person in the standard care arm was EUR 14 during the first year, assuming that 10% attended within one year for a check-up. Therefore, in the first year, the incremental detection costs were EUR 165 per screening participant. After this year, the model ignored the costs of repeat consultations including X-rays every 2–3 years, because these occurred in both the intervention and standard care arm (passive surveillance was removed in the intervention arm only for one year following screening, as mentioned in Section 2.1). Therefore, after the first year, there were no detection costs in the intervention arm, while in the standard care arm, detection costs were much reduced because they were limited to people diagnosed with lung cancer. See Table S6 in Supplementary File S1 for details on the detection costs; see Section 3.1 for the incremental detection costs over the lifetime horizon.

The costs included in relation to the false positives are shown in pink in Figure 2. In the model, a false positive was defined as any result that would require follow-up which in turn would reveal an absence of lung cancer. This included LDCT results that were either probably benign, suspicious or highly suspicious. For an LDCT scan, the probability of a false positive was based on an estimate by the International Agency for Research on Cancer [37]. This probability was adjusted by age using a previous study [38] based on data from the National Lung Screening Trial (NLST) in the United States [39], which found a higher probability of LDCT false positives among people aged 65–74 compared to those aged 55–64 [38]. For an X-ray, the probability of a false positive was based on the NLST [39]. This probability was not adjusted by age, based on a study that found similar odds of false positive results from chest radiography between people aged below 65 and 65 or older [40]. Overall, the cost of the false positives was higher in the screening arm compared to the standard care arm, because only a minority attended for a check-up visit in the standard care arm, and the probability of a false positive was lower with an X-ray than with an LDCT scan. See Table S8 in Supplementary File S1, and Supplementary File S11, for details.

The diagnostic and treatment pathways for people with lung cancer after the initial detection costs were not modelled explicitly: instead, the diagnostic and treatment costs for people with lung cancer were taken from a previous modelling study on LCS in the Italian context, which did not distinguish between diagnostic costs and treatment costs and calculated them over a five-year follow-up [13] (this source was selected in the absence of Italian longer-term data). They were applied only once per person in the model, at the time when people entered a diagnosed lung cancer state. These costs were stage-specific: the highest cost was for stage IV and the second-highest for stage III, although stage I was more expensive than stage II. See Table S7 in Supplementary File S1 for details. Screening affected the diagnostic and treatment costs by shifting the stage distribution to earlier stages.

### 2.4. Outcomes and Analysis

The primary outcome in the model was the incremental cost per quality-adjusted life year (QALY) gained. Unless otherwise specified, any mention of an incremental cost-effectiveness ratio (ICER) refers to the primary outcome. The incremental cost per QALY gained was assessed relative to a cost-effectiveness threshold of EUR 25,000. This was selected in the absence of an explicit cost-effectiveness threshold for decision-making in the health care sector in Italy [43]. A 2009 guideline proposal by the Italian Health Economics Association noted that health systems comparable to the Italian one used thresholds between EUR 25,000 and 40,000 per QALY gained, either implicitly or explicitly [44]. A threshold of EUR 25,000 was also used in a previous study on LCS among Italian smokers [13]. An analysis of 48 positive decisions on reimbursement made by the Italian Medicines Agency (AIFA) from 2016 to 2021 yielded an average of EUR 33,004 per QALY [43]. Another study on LCS among Italian smokers used a threshold of EUR 33,000 [14]. We considered that by using the lowest among all these thresholds, i.e., EUR 25,000, a conclusion of cost-effectiveness would then also apply to all other thresholds above EUR 25,000.

Other outcomes were the number of life years saved, the number of additional QALYs, the number of deaths averted, the incremental cost per life-year saved, the cost per death averted, overdiagnosis (defined as the proportion of people who are diagnosed with screening but would not have been diagnosed with standard care because they would have died from other causes before the cancer was diagnosed) and the number of surgeries in false positives (benign nodules). Note that the model did not distinguish between unnecessary and useful surgeries in false positives (sometimes a surgery for a benign nodule can be useful, for example, to make a diagnosis that leads to initiation of medical treatment or to remove a benign tumour that could grow in the near future).

Costs, life years and QALYs were half-cycle corrected and discounted by 3% (annual discount rate from [45]). The perspective was from the state-funded health care sector. The model was built in Microsoft^®^ Excel^®^ Version 2602 [46].

Parameter uncertainty was explored through multiple scenario analyses, listed below:

- Reducing overall screen-detectable prevalence among people aged 55–80 to a lower value reported in meta-analysis on people currently or formerly smoking exposed to asbestos [17].
- Changing the stage distribution at screening to a less favourable one, obtained from the baseline screening round of the DANTE trial conducted in Italy [47].
- Decreasing the utilities of people with lung cancer using a value which had been calculated for the United States in [48].
- Increasing the administrative and operating costs of screening per participant based on a model of LDCT LCS in Germany [24].
- Reducing the probability of a false positive result with an LDCT scan based on a study conducted in the United States [49].
- Changing the diagnostic and treatment costs of stages II to IV based on ratios vs. stage I taken from a study conducted in Italy [50].

Additionally, a scenario analysis was done to explore the effect of model simplifications relating to LC mortality after the 5th year since diagnosis and utilities. In this scenario analysis, it was assumed that after the 5th year since diagnosis, people no longer died from LC and had the same utility as people with no LC. See Table S9 in Supplementary File S1 for more details on the modifications made in the scenario analyses.

Probabilistic sensitivity analysis (PSA) based on 2000 iterations was conducted for the base case and for each scenario listed above. In each iteration, the analysis used values drawn by random sampling from the parameter distributions. Based on 2000 sets of results, the mean and 95% credible intervals (CIs) were calculated for each outcome.

## 3. Results

### 3.1. Results from the Base Case Analyses

Per 10,000 people screened, there would be 39 lung cancer deaths averted (95% CI: 13 to 79) and 481 life years saved (95% CI: 166 to 985), corresponding to 395 QALYs gained (95% CI: 129 to 831). Table 1 shows the results per screening participant, including an increase in total costs of EUR 109 per participant. However, there was a 9.2% probability that the intervention would be cost-saving rather than lead to a cost increase.

The average increase in total costs across all subgroups was given by the following components: an increase in detection costs (EUR 164 per screening participant); an increase in the cost of false positives (EUR 66 per screening participant); a saving in diagnosis and treatment costs (EUR-121 per screening participant). Reduced diagnostic and treatment costs with screening were due to the reduced costs of earlier stage diagnosis outweighing increased costs due to overdiagnosis and discounting.

**Figure 3:**
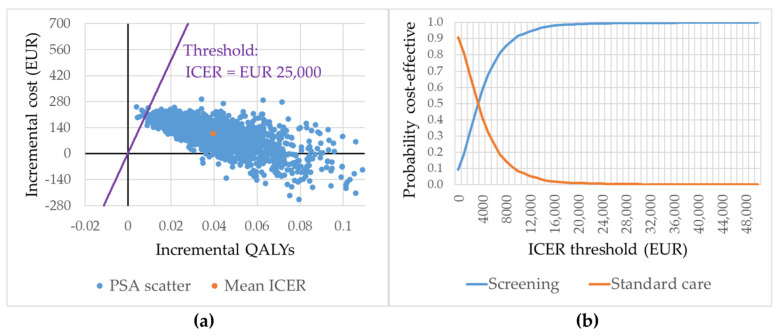
Distribution of PSA results on the cost-effectiveness plane and cost-effectiveness acceptability curve. Base case analysis, 2000 iterations. All subgroups together. (**a**) Distribution of PSA results on the cost-effectiveness plane. (**b**) Cost-effectiveness acceptability curve. Figure abbreviations: ICER: incremental cost-effectiveness ratio; QALY: quality-adjusted life year; PSA: probabilistic sensitivity analysis. The ICER refers to the incremental cost per QALY gained. See Supplementary File S13 for the probabilities of cost-effectiveness corresponding to different thresholds in (**b**).

Table 1 shows that across all subgroups, the incremental cost per additional QALY was EUR 2750. Figure 3a shows the distribution of PSA results on the cost-effectiveness plane. The figure indicates that the probability of cost-effectiveness relative to a threshold of EUR 25,000 was 99.5%. Figure 3b shows the probability of cost-effectiveness relative to other thresholds.

For all subgroups, on average, there was a gain in life years and QALYs arising from the intervention, with an increase in detection costs and the costs of the false positives and a saving in diagnosis and treatment costs (see Table 2). Females had bigger gains in life years than males. Because of this, in most age groups, females had higher QALY gains than males, despite their lower utilities. Females also had higher diagnostic and treatment savings. This was linked to their lower overdiagnosis, which in turn was connected to their lower probabilities of death from causes other than lung cancer. See overdiagnosis by subgroup in Table S25 in Supplementary File S14. The age group 55–59, which had the lowest screen-detectable prevalence, had the lowest gains in life years and QALYs. The same age group had the lowest saving linked to diagnosing and treating cancers at an earlier stage, and consequently, the highest total cost increase. Coherent with this, the age group with the highest screen-detectable prevalence (75–79) had the biggest savings in diagnosis and treatment costs and the lowest total cost increase. The age group 70–74 had the biggest gains in life years and QALYs.

The ICERs in Table 2 indicate that the intervention was cost-effective within each subgroup using a threshold of EUR 25,000 per QALY gained. See Supplementary File S14 for all results stratified by sex and age subgroup.

On average, the model estimated that, across all subgroups, per 10,000 people screened, 5 lung cancers would be found which would have never presented clinically if no screening had been done. Moreover, considering that the probability of a false positive with an LDCT was 18.0% on average, and the probability of surgery in a false positive was 0.8%, for every 10,000 people screened, there would be 14 surgeries on false-positive benign nodules.

### 3.2. Results from the Scenario Analyses

Across all scenario analyses, the intervention remained cost-effective in the model population aged 55 to 80. When screen-detectable prevalence was reduced, the ICER increased considerably, while more limited ICER changes occurred in the other scenario analyses. See Table 3 for the scenario analyses results.

When it was assumed that after the fifth year since a lung cancer diagnosis, utilities and mortality were the same as for people with no lung cancer, the ICER was slightly reduced because more life years and QALYs were gained from screening. Coherently with this, the ICER increased to a small extent when the lung cancer utilities were decreased.

## 4. Discussion

### 4.1. Summary of Key Results

This is the first cost-effectiveness study evaluating LCS among people exposed to asbestos within an Italian context. The model found that for those aged 55 to 80 with at least 10 pack-years and living in the ASUGI area, a single round of LDCT screening had a high probability of being cost-effective compared to standard care, relative to thresholds used in previous studies focusing on the Italian context (EUR 25,000 or higher) [13,14,43,44]. The intervention was cost-effective in the overall model population and it was also cost-effective across all sex and age subgroups included in the model.

If a lower cost-effectiveness threshold was used, the conclusion of cost-effectiveness would need to be re-assessed. There are different approaches to define cost-effectiveness thresholds for decision-making [51], which is a topic that goes beyond the scope of the paper. Further research should focus on how to define a cost-effectiveness threshold (or multiple thresholds linked to different criteria [51]) for the Italian state health care sector.

### 4.2. Strengths and Limitations

A strength of the model was that it combined parameters from the scientific literature with data from local registries and local costs. Moreover, local diagnostic pathways were incorporated into the calculation of detection costs and false-positive costs. When available, the model used parameters derived specifically from individuals with prior asbestos exposure who were currently or formerly smoking. However, due to data limitations, some parameters relied on information from populations without this dual exposure or living outside the ASUGI context and, in certain cases, outside Italy. In order to address this issue, another strength of the model was the use of scenario analyses to explore the effect of alternative model parameters or model simplifications on the results.

The scenario analyses confirmed the robustness of the finding from the base case analysis that the intervention was cost-effective relative to a threshold of EUR 25,000 in the overall model population aged 55 to 80. The ICER increased considerably in a scenario analysis that lowered screen-detectable prevalence to the level reported in [17], but the intervention remained cost-effective. Average screen-detectable prevalence in the model (0.0253) was higher than in other studies on people exposed to asbestos and smoking [17,22], which is line with a substantial percentage of older ages in the model population, based on the regional registry of people formerly exposed to asbestos [21]. Future screening programmes involving a sufficiently large number of participants should collect data on incidence by age.

One set of parameters that came with multiple limitations was the set of diagnostic and treatment costs for people with lung cancer. These costs had been calculated over a shorter time horizon (five years) than the model lifetime horizon, thus excluding any relapse and end-of-life care costs occurring from the sixth year since diagnosis. Moreover, they had been calculated in a different region and reported in a study published in 2020 [13], so it was not clear whether these costs fully applied within the ASUGI current context, due to the availability of new therapies and because ASUGI has established an LC diagnostic and treatment pathway developed at the local level [41]. Further research could estimate the lifetime diagnostic and treatment costs of lung cancer in the ASUGI context, using methods from the published literature [52,53,54,55].

Another set of parameters with considerable limitations was the set of utilities: they were estimated combining studies from Italy, England and the United States [27,28,29,30,31,32], and they were adjusted for smoking but not for asbestos exposure, so it was unclear whether they would apply to the model population. Additionally, utilities were affected by the absence of relapse and remission within the model structure.

While it is important to be aware of these limitations, scenario analyses using modified utilities or alternative diagnostic and treatment costs had a limited impact on the ICER, thus providing some reassurance on the robustness of the model results.

An additional strength of this research is that it is the first cost-effectiveness study of LCS within an Italian context to stratify the analysis by sex and age group. This stratification was important because various parameters changed by sex and age. However, the differences in results between subgroups should be interpreted with caution, for several reasons, including an assumption of equal screen-detectable prevalence between males and females; the use of a single age cut-off of 70 for the adjustment of the probabilities of death from LC during the first five years since diagnosis; the lack of age or sex adjustment for the probability of death from LC from the sixth year after diagnosis; the adjustment of LDCT false-positive probabilities based on a single age threshold of 65; and utility adjustments derived from only two age cut-offs (65 and 75).

The present work did not include the costs and benefits linked to incidental findings, such as the identification of interstitial lung disease, coronary artery calcification or extracoronary calcifications [56,57]. A review of economic models of LDCT LCS found that only a minority of studies included the costs of incidental findings, and none included the associated benefits [58]. However, the inclusion of incidental findings can have an important impact on cost-effectiveness results: an evaluation of upper abdominal CT screening in England, which included the benefits, costs and harms linked to the identification of ten cancers and abdominal aortic aneurysm, concluded that considering all relevant conditions is important to adequately capture the cost-effectiveness of screening interventions [59]. Therefore, future research should consider the inclusion of the health benefits, costs and potential risks and anxiety associated with follow-up examinations and interventions after incidental findings. The role of clinical protocols should be taken into account: a review noted that the management of incidental findings can have a considerable impact on cost-effectiveness [7].

Another limitation of this work is the lack of analysis of repeated screening. A UK-focused cost-effectiveness modelling study compared different screening frequencies, including a single screen and annual and biennial screening [60]. The study found that annual screening among people aged 55–80 with an LLPv2 risk score of >=3% was the most cost-effective option based on probabilistic sensitivity analysis [60]. The results from the UK model may not be applicable to the current model population and setting but suggest that further research may find better cost-effectiveness for annual or biennial screening than for the one-time screening evaluated here. Further research should look at repeated screening among people formerly exposed to asbestos within the Italian context and investigate the optimal frequency of screening, also considering personalised screening intervals based on different levels of risk [7].

### 4.3. Comparison with Previous Models

The current analysis has added evidence to two previous cost-effectiveness studies of LCS conducted in the Italian context [13,14]. The results were in line with these studies, which also found screening to be cost-effective. In contrast with the favourable results found in Italy, a previous modelling study found that biennial LDCT screening within the Canadian context was not cost-effective if targeted at people exposed to asbestos aged 55–74 with at least 10 pack-years, assuming a relative risk of 2 for lung cancer compared to people not exposed to asbestos [16]. Biennial screening became cost-effective if the minimum number of pack-years was increased to 15, but in both cases the incremental cost per additional QALY was considerably higher than in the evaluations focused on the Italian context, even after accounting for the different currency. The different results may be due to differences in parameters linked to the local context, such as mortality and costs [61].

### 4.4. Comments for Future Implementation and Research

A review on LCS highlighted that integrated smoking cessation interventions can improve the cost-effectiveness of LCS by reducing the risk of lung cancer and other diseases related to tobacco smoking [7]. This might improve cost-effectiveness even more for people who were exposed to asbestos and currently smoke, due to the synergistic effect of these risk factors [3]. Therefore, future economic evaluations could investigate this. Moreover, according to [19], LDCT for LCS should be read by radiologists educated in lung imaging and with specific skills in LCS. The benefits and costs of dedicated training have not been considered in the model and could be evaluated in future research. Furthermore, a review found that artificial intelligence (AI) could support radiologists in the detection of lung nodules and in the characterisation of nodules as benign or malignant [62]. Future research into the cost-effectiveness of LCS should take into account new developments in this field. At the same time, cost-effectiveness studies could support the evaluation of whether AI algorithms are reaching an acceptable balance between sensitivity and the probability of false positives.

The present evaluation was limited to a state-funded health care perspective because it was the most relevant perspective for local decision-makers. Further research could adopt a societal perspective, which enables the inclusion of all relevant societal costs and benefits associated with an intervention [63]. The scope of a societal perspective can vary [64], but examples of relevant costs are out-of-pocket healthcare costs, transport costs to attend healthcare visits, and productivity costs when paid or unpaid work is affected by illness or death [63,64,65,66]. Additionally, a societal perspective would typically consider the costs of informal caregiving or publicly or privately funded social care for people with lung cancer, as well as the quality of life of informal caregivers [63,65]. A societal perspective might affect subgroup results differently depending on whether they fall within the working-age range.

Further research could also employ equity-informative cost-effectiveness evaluation methods [67] that could quantify the equity effects of LCS among people exposed to both asbestos and smoking. For example, it could be taken into account whether people of higher socio-economic status are more likely to participate in screening or to benefit from it.

## 5. Conclusions

The model showed that, relative to thresholds of EUR 25,000 or higher mentioned in previous studies focusing on the Italian context [13,14,43,44], it was cost-effective to carry out a single round of LDCT LCS for people formerly exposed to asbestos with at least 10 pack-years of smoking, aged 55 to 80, and living in the ASUGI area. In particular, the intervention was cost-effective within each sex and age subgroup considered in the model. The model limitations should be taken into account when considering the conclusion of cost-effectiveness for policy-making. Various simplifications were made and some parameters came from populations living outside the ASUGI area and outside Italy or without dual exposure to smoking and asbestos. The scenario analyses provided some reassurance on the robustness of the model results within the model population aged 55 to 80, although these analyses were also conducted within a simplified modelling framework. The subgroup results should be interpreted with particular caution, as explained in the Discussion Section. Further research could include the costs and benefits of incidental findings, take a broader societal perspective, and assess the cost-effectiveness of periodic screening for the same population.

## Figures and Tables

**Table 1:** PSA incremental results (screening minus standard care) for life years, QALYs, deaths and total cost: weighted average across all subgroups. Mean and 95% CIs.

	Incremental LC Deaths	Incremental Life Years	Incremental QALYs	Incremental Total Cost (EUR)	ICERs		
					Incremental Cost (EUR) per LC Death Averted	Incremental Cost (EUR) per Life Year Saved	Incremental Cost (EUR) per Additional QALY
Mean	−0.0039	0.0481	0.0395	109	28,049	2260	2750
2.5th p	−0.0079	0.0166	0.0129	−85	NA	NA	NA
97.5th p	−0.0013	0.0985	0.0831	216	NA	NA	NA

Abbreviations: CI: credible interval; ICER: incremental cost-effectiveness ratio; LC: lung cancer; NA: not applicable; QALYs: quality-adjusted life years; p: percentile; PSA: probabilistic sensitivity analysis. Colour legend: light green = intervention more effective than comparator; amber = intervention more expensive than comparator; light blue = intervention cheaper than comparator. Results are per person participating in the screening programme and refer to a lifetime horizon. An ICER corresponds to the weighted average of incremental costs across all subgroups over the weighted average of incremental life years/deaths/QALYs across all subgroups.

**Table 2:** PSA mean incremental results (screening minus standard care) by subgroup: life years, QALYs and costs.

Subgroup	Life Years	QALYs	Detection Costs	Cost of False Positives	Diagnosis and Treatment Costs	Total Cost	ICER (Total Cost per QALY)
M 55–59	0.0229	0.0195	165	56	−46	175	8995
M 60–64	0.0283	0.0240	164	56	−61	159	6646
M 65–69	0.0492	0.0416	164	69	−111	122	2943
M 70–74	0.0585	0.0482	163	69	−145	88	1820
M 75–59	0.0529	0.0433	163	69	−148	84	1943
M 80	0.0341	0.0274	164	69	−103	129	4733
F 55–59	0.0237	0.0200	165	56	−46	175	8718
F 60–64	0.0299	0.0251	164	56	−63	158	6293
F 65–69	0.0520	0.0432	164	69	−114	119	2762
F 70–74	0.0597	0.0478	163	69	−153	80	1668
F 75–79	0.0547	0.0434	163	69	−162	70	1617
F 80	0.0356	0.0277	164	69	−116	117	4223

Abbreviations: F: female; ICER: incremental cost-effectiveness ratio; QALYs: quality-adjusted life years; M: males; PSA: probabilistic sensitivity analysis. Costs are in EUR. Colour legend: light green = intervention more effective than comparator; amber = intervention more expensive than comparator; light blue = intervention cheaper than comparator. Results are per person participating in the screening programme and refer to a lifetime horizon (with the exception of diagnostic and treatment costs, which had been calculated using a follow-up of only five years due to data availability limitations, as explained in Section 2.3.3).

**Table 3:** Scenario analyses, incremental mean results (screening minus standard care), weighted average across all subgroups.

Analysis	Life Years	QALYs	Detection Costs	Cost of False Positives	Diagnosis and Treatment Costs	Total Cost	ICER: Cost per QALY
Base case	0.0481	0.0395	164	66	−121	109	2750
Reduced overall screen-detectable prevalence	0.0181	0.0140	165 ^1^	66	−45	185	13,270
Less favourable stage distribution at screening	0.0351	0.0288	164	66	−77	153	5312
Lower utilities for people with lung cancer	0.0481	0.0302	164	66	−121	109	3601
After the 5th year of diagnosed LC, the same mortality and utility as for people with no LC were applied.	0.0634	0.0525	164	66	−121	109	2068
Higher administrative and operating costs of screening	0.0481	0.0395	180	66	−121	125	3161
Reduced probability of a false positive with LDCT	0.0481	0.0398	164	48	−121	90	2264
Different diagnostic and treatment costs for stages II–IV	0.0481	0.0395	164	66	−99	131	3304

Abbreviations: ICER: incremental cost-effectiveness ratio; LC: lung cancer; LDCT: low-dose computed tomography; QALY: quality-adjusted life year. Costs are in EUR. Colour legend: light green = intervention more effective than comparator; amber = intervention more expensive than comparator; light blue = intervention cheaper than comparator. Results are per person participating in the screening programme and refer to a lifetime horizon (with the exception of diagnostic and treatment costs, which had been calculated using a shorter follow-up due to data availability limitations). ^1^ When the screen-detectable prevalence was reduced, the detection costs were the same in the screening arm, but they decreased in the standard care arm, so the incremental detection costs were higher.

## Data Availability

Restrictions apply to the availability of data from the FVG registry of people formerly exposed to asbestos [21]. These data are available from the registry. Restrictions apply to the availability of data on lung cancer incidence by age group in the Trieste province. These data were obtained from the FVG cancer registry [82]. Data from the FVG cancer registry are available under restricted access due to patient confidentiality and privacy concerns. Access can be obtained upon request to the Director of the Friuli Venezia Giulia Cancer Registry and to the Director of SC Pianificazione, Programmazione e Controllo Direzionale, Azienda Regionale di Coordinamento per la Salute, Udine, Italy. Due to these restrictions, the Excel^®^ model is not available. Other data used in this study were derived from published studies listed in the References Section. Some of these data are presented in the Supplementary Materials. The Supplementary Materials also show the probabilities of death from lung cancer extracted from the FVG cancer registry [25]. Further inquiries can be directed to the corresponding author.

## Supplementary Materials

The following supporting information can be downloaded at: https://www.mdpi.com/article/10.3390/jcm15083136/s1. References [68,69,70,71,72,73,74,75,76,77,78,79,80,81] are cited in the Supplementary Materials.

## Abbreviations

The following abbreviations are used in this manuscript:

AI	Artificial intelligence
ASUGI	Azienda Sanitaria Universitaria Giuliano Isontina
BCA	Base case analysis
CI	Confidence interval for parameters; credible interval for results
CT	Computed tomography
DLCO	Diffusing capacity of the lungs for carbon monoxide
FVG	Friuli Venezia Giulia
HICP	Harmonised Index of Consumer Prices
HRCT	High resolution computed tomography
ICER	Incremental cost-effectiveness ratio
INCR	Incremental
INT	Intervention
LC	Lung cancer
LCS	Lung cancer screening
LDCT	Low-dose computed tomography
LL	Lower limit
LLPv2	Version 2 of the Liverpool Lung Project risk score
MRI	Magnetic resonance imaging
NA	Not applicable
NLST	National Lung Screening Trial
NMB	Net monetary benefit
P	Probability or proportion for parameters; percentile in the results
PET	Positron emission tomography
PSA	Probabilistic sensitivity analysis
QALY	Quality-adjusted life year
RCT	Randomised controlled trial
SA	Scenario analysis
SC	Standard care
SD	Standard deviation
UK	United Kingdom
UKLS	United Kingdom Lung Screen
UL	Upper limit
YR	Year
WA	Weighted average
