# Supplementary material for: Lung Cancer Screening in a Population from Northeast Italy Exposed to Both Asbestos and Smoking: A Cost-Effectiveness Analysis
Source: J Clin Med. 2026 Apr 20;15(8):3136. doi: 10.3390/jcm15083136 (PMC13117614; doi:10.3390/jcm15083136)
Supplement: Supplementary file 1 [file jcm-15-03136-s001.zip › jcm-4196287-supplementary.pdf]

Supplementary Files S1–S14

Lung cancer screening in a population of Northeast Italy exposed to both asbestos and smoking: a cost-effectiveness analysis

Supplementary File S1. Details on model parameters

Subgroup proportions (proportions of males and females, and proportions within each age subgroup) were calculated using [21] and are not reported due to restrictions on data availability – see data availability statement. A beta distribution was used for the proportion of males. For the proportions within each subgroup, a Dirichlet distribution was used.

Table S1. Input parameters, base case – screen-detectable prevalence, stage distribution, and probabilities applied to undiagnosed lung cancers.

Parameter	Mean	95% CI and distribution	Source
**Screen-detectable prevalence**[1]			
Age group 55-59	0.0086	NA, linked to other parameters, see Supplementary File S3.	[22] combined with [68] for calculation. See Supplementary File S3.
Age group 60-64	0.0116		
Age group 65-69	0.0216		
Age group 70-74	0.0301		
Age group 75-79	0.0344		
Age 80	0.0266		
**Stage distribution with screening**			
Stage I	0.64	NA[2]	[9]
Stage II	0.19		
Stage III	0.12		
Stage IV	0.05		
**Stage distribution at diagnosis with standard care**			
Stage I	0.19	NA[2]	[23]
Stage II	0.12		
Stage III	0.29		
Stage IV	0.40		
**3-month probabilities of progressing to higher undiagnosed lung cancer stages**			
Stage I to stage II	0.3558	NA[2]	[24]
Stage I to stage III	0.0328		
Stage I to stage IV	0.0869		
Stage II to stage III	0.2540		
Stage II to stage IV	0.1290		
Stage III to stage IV	0.1048		
**3-month probabilities of diagnosis for undiagnosed lung cancer**			
Stage I	Depends on sex and age, see Supplementary File S5	NA[3]	Calculated based on other parameters, see Supplementary File S5
Stage II			
Stage III			
Stage IV	0.6584	NA[4]	[24]

Table notes. Abbreviations: CI: confidence interval; NA: not applicable.

[1]Overall, combining the point estimates of screen-detectable prevalence for each age subgroup with the point estimate of the proportion in each sex and age subgroup, the screen-detectable prevalence was 2.53%.

[2] NA: Only a deterministic point estimate was used for parameters that were included for the iterative calculation of the probability of diagnosis from undiagnosed stage I, II and III. This was done in order to simplify the iterative calculation. Details about the calculation are in Supplementary File S5.

[3] In order to simplify the iterative calculation, only the point estimates of the probabilities of diagnosis were calculated. Details about the calculation are in Supplementary File S5.

[4] NA: Only the point estimate was used because there was no data available regarding the 95% CI or the distribution.

Table S2. Input parameters relating to the probabilities of death, base case.

Parameter	Mean	95% CI and distribution	Source
**Ratios comparing the 3-month P of death from LC between different stages**			
SII vs SI, first year since diagnosis	2.0	NA, linked to beta distributions for the annual probabilities, which in turn, are based on the numbers reported in [23].	[23]
SIII vs SI, first year since diagnosis	4.7		
SIV vs SI, first year since diagnosis	10.3		
SII vs SI, second year since diagnosis	2.4		
SIII vs SI, second year since diagnosis	4.4		
SIV vs SI, second year since diagnosis	6.9		
SII vs SI, third year since diagnosis	1.5		
SIII vs SI, third year since diagnosis	2.1		
SIV vs SI, third year since diagnosis	3.5		
SII vs SI, fourth year since diagnosis	2.0		
SIII vs SI, fourth year since diagnosis	2.8		
SIV vs SI, fourth year since diagnosis	2.7		
SII vs SI, fifth year since diagnosis	1.3		
SIII vs SI, fifth year since diagnosis	1.8		
SIV vs SI, fifth year since diagnosis	2.5		
**3-month probabilities of death from lung cancer**			
3-month P of death from LC during the first 5 years since diagnosis: the probability changed by stage at diagnosis, year since diagnosis, sex and age. More details are provided in Table S13 in Supplementary File S7.		NA. Linked to other parameters. See Supplementary Files S6 – S7 for more details.	[23,25,69]. See Supplementary Files S6 – S7 for details on the calculation.
3-month P of death from LC after the fifth year since diagnosis, all stages, all ages, both sexes	0.013	NA[1]	[26]. See Supplementary File S7.
**3- month probability of death from causes other than lung cancer**			
The probability changed by age and sex, see Supplementary File S8 for details		NA[2]	[70-73] were combined for calculation. See Supplementary File S8 for details.

Table notes. Abbreviations: CI: confidence interval; NA: not applicable.

[1] NA: Only the point estimate was used because there was no data available regarding the 95% CI or the distribution.

[2] NA: Only a deterministic point estimate was used for parameters that were used for the iterative calculation of the probability of diagnosis from undiagnosed stage I, II and III. This was done in order to simplify the iterative calculation.

Table S3. Input parameters, base case – utilities for people with no lung cancer and for different stages of lung cancer.

Parameter	Mean	95% CI and distribution	Source
No LC, males, 55-64	0.925	NA, linked to other parameters, see Supplementary File S9.	The calculation combined the utilities reported in [27-29], applying weights from [21,22]. See Supplementary File S9 for details.
No LC, males, 65-74	0.899		
No LC, males, 75+	0.847		
No LC, females, 55-64	0.910		
No LC, females, 65-74	0.896		
No LC, females, 75+	0.811		
Stage I, males, 55-64	0.913	NA, linked to other parameters, see Supplementary File S9.	For the estimation, we calculated a ratio comparing utilities in the absence and presence of lung cancer, using sources listed in the row above as well as [30]. Utilities for different stages of lung cancer were estimated using [30,31]. See Supplementary File S9.
Stage I, males, 65-74	0.887		
Stage I, males, 75+	0.836		
Stage I, females, 55-64	0.899		
Stage I, females, 65-74	0.885		
Stage I, females, 75+	0.801		
Stage II, males, 55-64	0.868		
Stage II, males, 65-74	0.844		
Stage II, males, 75+	0.795		
Stage II, females, 55-64	0.854		
Stage II, females, 65-74	0.841		
Stage II, females, 75+	0.761		
Stage III, males, 55-64	0.868		
Stage III, males, 65-74	0.844		
Stage III, males, 75+	0.795		
Stage III, females, 55-64	0.854		
Stage III, females, 65-74	0.841		
Stage III, females, 75+	0.761		
Stage IV, males, 55-64	0.857		
Stage IV, males, 65-74	0.833		
Stage IV, males, 75+	0.785		
Stage IV, females, 55-64	0.843		
Stage IV, females, 65-74	0.830		
Stage IV, females, 75+	0.751		

Table notes. Abbreviations: CI: confidence interval; LC: lung cancer; NA: not applicable. Colour legend: light blue: males; amber: females. Note that as people aged through the model years, we also used weighted averages of the utilities presented above: for example, males aged 60-64 at model entry were aged 61-65 in the second model year, so during that year, the average utility with stage IV for that subgroup was 0.852, which was a weighted average of the stage IV utilities for males aged 55-64 and 65-74 (in this example, the weights were the number of males residents in the Friuli Venezia Giulia region in 2023 within each single age, taken from [74]).

Table S4. Input parameters, base case – utilities linked to screening and false positives.

Parameter	Mean	95% CI		Distribution	Distribution parameters		Source
		LL	UL		Mean / shape	SD / scale	
Disutility for anxiety of screening event	-0.01	-0.011	-0.009	Normal	-0.010	0.00028	[32,75]
Disutility for a false positive	-0.063	-0.074	-0.052	Normal	-0.063	0.00560	[32,76]
Duration of disutility from screening anxiety in years	0.04	0.010	0.084	Gamma	4	0.0096	[32]
Duration of disutility for a false positive in years	0.10	0.026	0.209	Gamma	4	0.0238	Assumption[1]
QALY loss from screening anxiety	-0.00038	NA, linked to parameters above					
QALY loss from false positive	-0.0060	NA, linked to parameters above					

Table notes. Abbreviations: CI: confidence interval; LL: lower limit; NA: not applicable; SD: standard deviation; UL: upper limit.

[1] The average duration was assumed to be one month for people with only a follow-up HRCT, and three months for people with additional investigations. Using the probability of having additional investigations vs. not having them in the screening arm, an average duration was estimated.

Table S5. Inflation indices

Inflation index	Annual % change	Source
The Harmonized Index of Consumer Prices (HICP) for hospital services, Italy, 2002-2023	0.9	[35]
The Harmonized Index of Consumer Prices (HICP) for outpatient services, Italy, 2001-2023	2.1	[36]

Table S6. Input parameters, base case – detection costs (initial screening and passive surveillance costs).

Parameter	Mean	95% CI		Distribution	Distribution parameters		Source
		LL	UL		Shape	Scale	
**Initial screening costs**							
Admin & operating costs of screening per participant	18.5	15.0	22.3	Gamma	100	0.18	Inflated from [13][1]
Cost of initial visit to discuss risks and benefits	31.5	25.6	38.0	Gamma	100	0.32	Inflated from [33][1]
Cost of LDCT scan	129.4	105.3	156.0	Gamma	100	1.29	Inflated from [33][1]
Initial cost of screening per participant	179.4[2]	NA, linked to parameters above					Parameters above
**Initial passive surveillance costs**							
P (control visit) in one year in standard care arm	0.10	NA. Only the point estimate was used because there was no data available regarding the 95% CI or the distribution.					Assumption
Cost of a visit	31.5	25.6	38.0	Gamma	100	0.32	Inflated from [33][1]
Cost of X-Ray	27.3	22.2	32.9	Gamma	100	0.27	Inflated from [33][1]
Cost of global spirometry	51.5	41.9	62.1	Gamma	100	0.52	Inflated from [33][1]
Cost of DLCO	32.2	26.2	38.8	Gamma	100	0.32	Inflated from [33][1]
Cost of a control visit + tests above in the standard care arm	142.5	NA, linked to parameters above					Parameters above

Table notes. Abbreviations: CI: confidence interval; DLCO: diffusing capacity of the lungs for carbon monoxide; LC: lung cancer; LDCT: low-dose computed tomography; LL: lower limit; NA: not applicable; UL: upper limit. Costs in EUR.

[1] See Supplementary File S12 for costs reported in the relevant sources, before they were inflated. When relevant, the same Supplementary File also reports the cost code used in official sources.

[2] Cost per 10,000 participants: 1,794,114.

Table S7. Input parameters, base case – diagnostic and treatment costs for people with lung cancer.

Parameter	Mean	95% CI		Distribution	Distribution parameters		Source
		LL	UL		Alpha / shape	Beta / scale	
Cost of workup and treatment, stage IA	30,451	24,776	36,702	Gamma	100	305	Inflated from [13][1]
Cost of workup and treatment, stage IB	29,093	23,671	35,065	Gamma	100	291	Inflated from [13][1]
P (stage IA) if stage I	0.48	0.428	0.526	Beta	187	205	[23]
P (stage IB) if stage I	0.52	NA, linked to parameter above					
Cost of workup and treatment, stage I	29,741	NA, linked to parameters above					
Cost of workup and treatment, stage II	24,750	20,138	29,831	Gamma	100	248	Inflated from [13][1]
Cost of workup and treatment, stage III	36,233	29,481	43,672	Gamma	100	362	Inflated from [13][1]
Cost of workup and treatment, stage IV	42,450	34,539	51,165	Gamma	100	425	Inflated from [13][1]

Table notes. Abbreviations: CI: confidence interval; LL: lower limit; NA: not applicable; UL: upper limit. Costs in EUR.

[1] See Supplementary File S12 for costs reported in the relevant sources, before they were inflated.

Table S8. Costs of false positives, base case

Parameter	Mean	95% CI		Distribution	Distribution parameters		Source
		LL	UL		Alpha/ shape	Beta / scale	
P (FP) if LDCT	0.180	0.157	0.204	Beta	180	820	[37]
Ratio comparing the probability of FPs at ages 65+ vs ages <65	1.221	Linked to beta distributions based on numbers and probabilities reported in [38]					[38]
P (FP) if LDCT at ages <65	0.154	Linked to the ratio above and to probabilities of being aged <65 or >=65 (reported in [21])					[21,38]
P (FP) if LDCT at ages 65+	0.188						
P (FP) if X-ray	0.086	0.083	0.090	Beta	2251	23784	[39]
P (FP) if in SC arm	0.009	NA, linked to parameters above					
N of HRCTs if HRCT	1.00	NA, assumption for mean only; no assumption was made for the 95% CI or the distribution.					
Cost of HRCT (no contrast agent)	129.4	105.3	156.0	Gamma	100	1.29	Inflated from [33][1]
P (additional investigations) if FP with LDCT	0.072	0.039	0.114	Beta	13	167	[37]
P (additional investigations) if FP with X-ray	0.015	0.012	0.019	Beta	70	4604	[39]
Cost of additional investigations after a positive HRCT	2307.8	NA, sum of other cost parameters, which were taken from [33][1]; inflation applied (see Supplementary File S12 for uninflated parameter).					
P (surgery) if FP with LDCT	0.008	0.002	0.018	Beta	4	491	[77]
P (surgery) if FP with X-ray	0.010	0.007	0.013	Beta	45	4629	[39]
Cost of surgery	10,572	8,602	12,743	Gamma	100	105.72	Inflated from [34][1]
Cost of one FP in the screening arm	381.5	NA, linked to parameters above					
Cost of FPs per screening participant if aged <65	58.6	NA, linked to the cost of one FP and the P of FPs with LDCT at ages <65					
Cost of FPs per screening participant if aged >=65	71.6	NA, linked to the cost of one FP and the P of FPs with LDCT at ages >=65					
Cost of one FP in the standard care arm	265.4	NA, linked to parameters above					
Cost of FPs per person in the standard care arm	2.3	NA, linked to: cost of one FP; P of one control visit in the standard care arm; P of an FP with an X-ray.					

Table notes. Abbreviations: CI: confidence interval; FP: false positive; LL: lower limit; NA: not applicable; P: probability; UL: upper limit. Costs in EUR.

[1] See Supplementary File S12 for costs reported in the relevant sources, before they were inflated. When relevant, the same Supplementary File S12 also reports the cost code used in official sources.

Table S9 describes the scenario analyses where specific parameters were modified to see how results would change.

Table S9. Scenario analyses: descriptions and justifications.

**Modified parameters**	**Justification for scenario analysis (SA) and parameter modification compared to the base case analysis (BCA).**
Screen-detectable prevalence	There was uncertainty around the assumptions made for the calculation of this parameter (see Supplementary File S3). In the overall population aged 55-80, on average, screen-detectable prevalence was 0.0253 (by age group: 55-59: 0.0086; 60-64: 0.0116; 65-69: 0.0216; 70-74: 0.0301; 75-79: 0.0344; 80: 0.0266). In a SA, overall screen-detectable prevalence was changed to 0.0094. This value was taken from a meta-analysis of 12 studies on LDCT LCS in people currently or formerly smoking and exposed to asbestos [17]. For simplification, only the point estimate was used from [17]. Screen-detectable prevalence within each age group was lowered accordingly, to these values, on average: 55-59: 0.0032; 60-64: 0.0043; 65-69: 0.0080; 70-74: 0.0112; 75-79: 0.0128; 80: 0.0099. These values varied probabilistically because they were linked to the beta distributions used for incidence (incidence was used to estimate age variation in screen-detectable prevalence).
Stage distribution at screening	The BCA screening distribution was taken from the UKLS trial [9], and there was uncertainty around its applicability to the model population. In a SA, a less favourable distribution from the baseline screening round of the DANTE trial [47] was applied (The two distribution differed as follows: UKLS: SI: 0.643, SII: 0.190, SIII: 0.119, SIV: 0.048 [9]. DANTE: SI: 0.571, SII: 0.107, SIII: 0.179, SIV: 0.143. [47]).
3-month probability of death from LC and utility after the 5th year of diagnosed LC	In the BCA, the 3-month probability of death from LC after the 5th year since diagnosis 0.013, and the stage-specific utility was the same during each year from diagnosis until death, which was a model simplification. In a SA, it was assumed that after the 5th year, people no longer died from LC, and they had the same utility as people with no LC.
Utilities for people with LC	In the BCA, the overall utility for people with LC (referring to both sexes, all ages and all stages) was 0.850 (95% CI: 0.829 to 0.871), based on an Italian study [30]. However, it was considered that people with LC who have been exposed to both asbestos and smoking may have lower utilities than other people with LC. In a SA, the overall utility for people with LC was lowered to 0.719 (95% CI: 0.678 to 0.760), as per the United States EQ-5D-3L utility for cancer of bronchus and lung, reported in [48]. The SA age-and-sex-specific utilities were then decreased accordingly.
Administrative and operating costs of screening	There was uncertainty around these costs because screening had not yet been implemented. In the BCA, the costs were EUR 18.5 (95% CI: 15.0 to 22.3) per screening participant. In a SA, the costs were increased to EUR 34.70 (95% CI:

Modified parameters	Justification for scenario analysis (SA) and parameter modification compared to the base case analysis (BCA).
	28.2 to 41.8). The SA parameter was taken (in its inflated-to-2023 version) from a model on LDCT LCS in Germany [24], which reported EUR 30 in 2016-related costs.
Probability of an FP result with an LDCT scan	The BCA age-specific probabilities were calculated starting from this BCA parameter: 0.180 on average, 95% CI: 0.157 to 0.204. This was originally calculated in relation to screening repeated 3 times with annual intervals [37]. The estimation assumed that the Lung-RADS protocol (version 1.0) was applied to interpret the scans [37]. However, the model focused on a one-off screening and ASUGI aimed to implement the latest version of the Lung-RADS protocol rather than the older 1.0 version. Therefore, there was uncertainty around the BCA parameter. In a SA, the proportion was lowered to 0.131 (95% CI: 0.123 to 0.140; beta distribution with parameters: alpha=766; beta: 5069), as per [49]. (The age-specific probabilities decreased accordingly).
Diagnostic and treatment costs of stage II, III and IV	There was uncertainty on whether the diagnostic and treatment costs calculated based on a hospital in the Lombardy region for a model with a 5-year time horizon published in 2020 [13] would apply to the ASUGI current context and to the model lifetime horizon. Therefore, different costs of SII, SIII and SIV were used in a SA. More specifically, the SA used ratios of the costs of SII, SIII and SIV vs. SI reported in a study conducted in the Veneto region in Italy [50]: 1.19, 1.34, 1.36, respectively. The study [50] estimated these ratios based on costs incurred only during the first year since the lung cancer was first suspected, so it was not fully applicable to the model lifetime horizon, but it was used for exploratory purposes. Based on these ratios, the costs in the SA were: SII: EUR 35,391 (95% CI: 28,796 to 42,657); SIII: EUR 39,853 (95% CI: 32,426 to 48,034); SIV: EUR 40,447 (32,910 to 48,751).<br><br>The costs of SII, SIII and SIV in the BCA are reported in Table S7. In the BCA, the ratios of the costs of SII, SIII and SIV vs. SI were, on average: 0.83, 1.22, 1.43. So in the SA, compared to the BCA, SII and SIII were more expensive, and SIV less expensive.

Table notes. Abbreviations: ASUGI: Azienda Sanitaria Universitaria Giuliano Isontina; BCA: base case analysis; FP: false positive; IARC: International Agency for Research on Cancer; LC: lung cancer; LDCT: low-dose computed tomography; SA: scenario analysis.

Supplementary File S2. More details on the model structure

Not modelling false negatives

False negatives were not modelled because the same set of long-term outcomes was expected for false negative results in the intervention arm as for lung cancers in the standard care arm. Moreover, at the start of the model, the proportion with lung cancer in the standard care arm was equal to the proportion with lung cancer in the screening arm, so by ignoring false negatives in the screening arm, the proportion with lung cancer also became smaller in the standard care arm. Therefore, incremental results were not affected by the non-modelling of false negatives. Due to this simplification, the model does not provide the overall cost of lung cancer in the presence or in the absence of screening. The model can only be used to estimate the incremental life years, incremental QALYs and incremental cost associated with screening (the term "incremental" refers to the difference in outcomes between the screening arm and the standard care arm).

Markov model: intervention arm

The Markov model for the intervention arm did not include undiagnosed lung cancer. See figure S1.

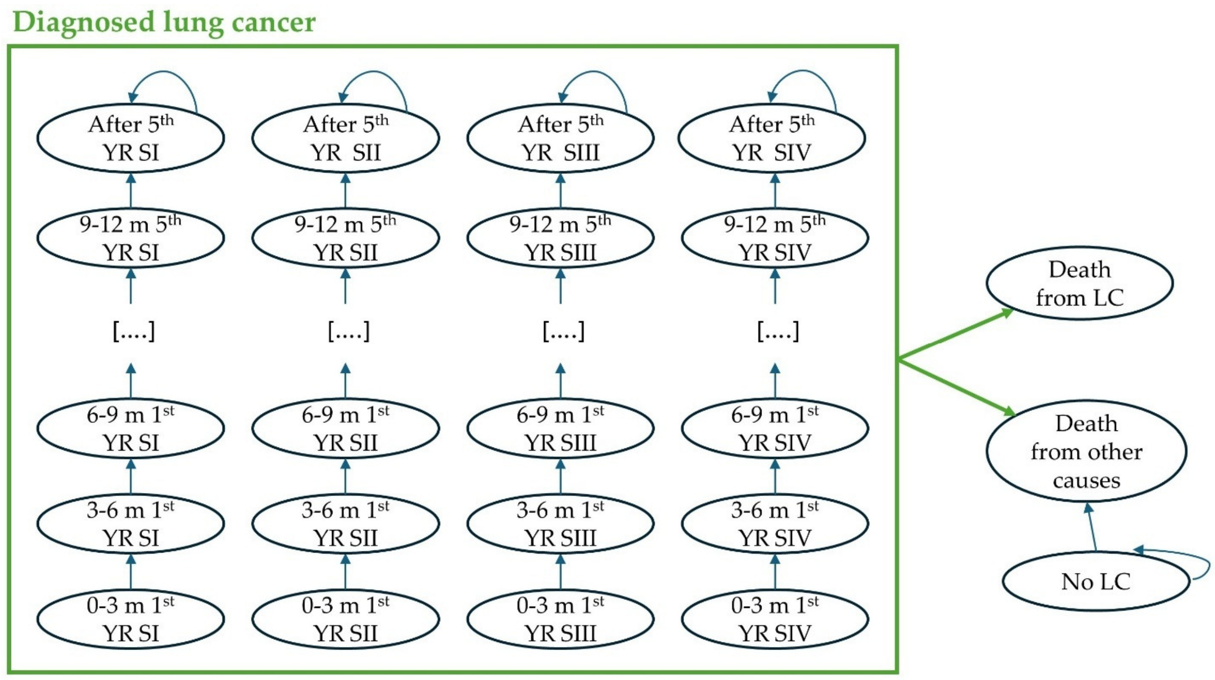

Figure S1. Markov model, intervention arm.

Figure notes. Each circle is a different "health state". People spend 3 months in each health state, with these exceptions: 1. people can stay for more than 3 months in states where there is an arrow that re-turns to the same circle; 2. death states (so-called "absorbing states"). The green outline indicates diagnosed lung cancer. From any of the diagnosed cancer stages, people can transition to death from LC or to death from other causes.

Abbreviations: LC: lung cancer; SI: stage I; SII: stage II; SIII: stage III; SIV: stage IV; YR: year.

Supplementary File S3. Details on estimation of screen-detectable prevalence.

Screen-detectable prevalence by sex.

Due to a lack of data on how screen-detectable prevalence may differ between men and women in a population formerly exposed to asbestos with at least 10 pack-years of smoking, the model assumed that screen-detectable prevalence was the same for men and women. This was a model simplification made in light of limited evidence on the topic, summarized as follows: according to a systematic review, smoking produces sim-ilar risks of lung cancer in men and women; potentially, women may be at higher risk than men, but this is not yet clear from the available data [78]. Another review [79] found that after adjusting for smoking, age, study, and occupations which would involve high exposure to other carcinogens, the OR for lung cancer associated with occupational as-bestos exposure was 1.24 for men (95% CI: 1.18 to 1.31) and 1.12 for women (95% CI: 0.95 to 1.31) [79]. Note the overlapping confidence intervals.

Against the assumption of equal screen-detectable prevalence, the same review [79] found a lower median cumulative exposure level to asbestos for exposed women than for exposed men. Moreover, in a study on people formerly exposed to asbestos in Italy [73], 28% of women were wives of asbestos-cement workers, which means that they had secondhand exposure.

Screen-detectable prevalence is influenced not only by the underlying lung cancer risk but also by the screening intervention. It was assumed that the screening intervention and the diagnostic pathways were uniform across sexes.

In conclusion, despite potential differences between men and women, given a lack of data on how screen-detectable prevalence may differ by sex, it was decided to use the same screen-detectable prevalence for men and women.

Screen-detectable prevalence by age group.

A study conducted within the ASUGI area [22] found that with a baseline round of LDCT screening, the proportion diagnosed with lung cancer (i.e., the screen-detectable prevalence) was 0.01205 among people with at least 10 pack-years of smoking exposed to asbestos (6 lung cancers were identified among 498 people). The study did not provide age and sex information specifically for the 498 people with at least 10 pack-years but it was mentioned that the wider study population of 1045 people was aged 44-75 and 97% of these 1045 people were men.

In our model, in the absence of relevant data, variation by age in screen-detectable prevalence mirrored variation by age in incidence in the general population of Trieste province with standard care (average annual incidence for 2017-2021 from the FVG cancer registry [68]). For example, people aged 70-74 had a screen-detectable prevalence that was 1.39 times the screen-detectable prevalence of people aged 65-69, which mirrored the ratio that compared incidence between these two age groups. This was obtained by first calculating a risk ratio between screen-detectable prevalence in [22] and overall incidence in the age group 45-74 [68]. (Note the slight age range discrepancy between 44-75 in [22] and 45-74: the reason for this was that incidence data from [68] was reported in 5-year age groups, such as 45-49, 50-54, 70-74). Then incidence in each age group was multiplied by this risk ratio to obtain screen-detectable prevalence by age group. A beta distribution was used for incidence. Incidence is not reported here due to restrictions on data availability, see data availability statement. Note that these calcula-tions used these starting points:

1. the weighted average of the screen-detectable prevalence among people aged 44-75, exposed to asbestos and with at least 10 pack-years, was assumed to be the same in the ASUGI area in 2025 as in Monfalcone in 2002-2003 (which was the context of [22]): 0.01205, as long as the age distribution in the population of interest would be similar as it was in [22], as would anything else contributing

to the screen-detectable prevalence in the two populations. Moreover, since the age range in [22] referred to the overall group of 1045 participants, another assumption was that the age of 498 people with at least 10 pack-years in [22] was similar to the age of the overall group of 1045 participants.

2. it was assumed that undiagnosed prevalence is similar in its age distribution to incidence, and screening sensitivity doesn't vary by age.

Table S10. Screen-detectable prevalence in [22].

	**Point estimate**	**Distribution**
**People of age 44-75 exposed to asbestos and who had at least 10 pack-years of smoking who received LDCT screening in Monfalcone in 2002-2003** [22]		
N people	498	NA
N lung cancers	6	NA
Proportion diagnosed	0.01205	Beta distribution. Parameters: alpha: 6; beta: 492
Proportion diagnosed per 100,000	1205	Linked to distribution above

Table notes. Abbreviations: LDCT: low-dose computed tomography; N: number; NA: not applicable.

The proportion diagnosed in Table S10 was combined with incidence by age group to produce the screen-detectable prevalence by age group (shown in Table S1). Probabilistic sampling was linked to the beta distributions for incidence and the beta distribution in Table S10.

Supplementary File S4. More details on using the stage distribution at screening from the UKLS trial.

The stage distribution for the screening arm was taken from the UKLS one-off screening [9]. This was preferred over taking the distribution from the baseline screening in the ITALUNG trial [80] because the UKLS distribution was based on 42 cancers, while the ITALUNG baseline distribution was only based on 18 cancers. (The two distribution differed as follows: UKLS: SI: 0.64, SII: 0.19, SIII: 0.12, SIV: 0.05 [9]. ITALUNG: SI: 0.56, SII: 0.11, SIII: 0.11, SIV: 0.22. The latter distribution excluded two people with small cell lung cancer [80]).

To check if it was valid to use the stage distribution from the screening arm of UKLS, the stage distribution in the standard care arm of UKLS was compared to the stage distribution used for the model standard care arm, which was taken from a study conducted in Italy based on 2050 cancers [23]. Table S11 shows that the UKLS standard care arm had more cancers diagnosed in stage IV and fewer cancers diagnosed in stage III, compared to [23]. It was noted that the stage distribution from the standard care arm of the ITALUNG trial differed even more from [23]. All considered, it was decided to use the distribution from the screening arm of UKLS.

Table S11. Validation check. Comparing the distribution in the standard care arm of the trials UKLS and ITALUNG to the distribution in [23]

**Stage**	**Proportion diagnosed in each stage with standard care**		
	**UKLS** [9]	**Consonni et al.** [23]	**ITALUNG** [81]
SI	0.22	0.19	0.14
SII	0.11	0.12	0.09
SIII	0.18	0.29	0.14
SIV	0.49	0.40	0.63
SUM	1.00	1.00	1.00

Table notes. The distribution in the non-screening arm of the ITALUNG trial referred to annual screening over 4 years and was based on 56 cancers [81].

Supplementary File S5. Probability of diagnosis from undiagnosed stage I, II, III

The probabilities of diagnosis from undiagnosed stage I, II and III were calculated through iterative calculation based on the stage distribution of undiagnosed lung cancer at the start of the model (equal to the distribution at screening), the stage distribution at diagnosis in the standard care arm, the 3-month probability of progressing to higher stages, and the 3- month probability of death from causes other than lung cancer, different for each age and sex subgroup. The iterative calculation was only done with deterministic point estimates for simplification. As a result, the probabilities of diagnosis from undiagnosed stage I, II and III were the point estimates shown in in Table S12, which did not change probabilistically. Note that the probabilities decreased for older age groups because mortality from other causes was higher at older ages. However, for each subgroup, the probability stayed constant across time. This was a model simplification.

Table S12. 3-month probability of diagnosis, by stage and subgroup, base case. Based on iterative calculations.

Subgroup	3-month probability of diagnosis if undiagnosed		
	Stage I	Stage II	Stage III
Males 55-59	0.201	0.118	0.277
Males 60-64	0.200	0.118	0.277
Males 65-69	0.199	0.117	0.277
Males 70-74	0.198	0.117	0.276
Males 75-79	0.196	0.116	0.275
Males 80	0.194	0.115	0.274
Females 55-59	0.201	0.118	0.277
Females 60-64	0.201	0.118	0.277
Females 65-69	0.200	0.118	0.277
Females 70-74	0.199	0.117	0.277
Females 75-79	0.198	0.117	0.276
Females 80	0.196	0.116	0.275

The probability of remaining in the same undiagnosed stage at the end of a three-month cycle was dependent on the probability of progressing to a higher undiagnosed stage (see Table S1 in Supplementary File S1), the probability of diagnosis (see paragraphs above) and the probability of mortality from causes other than lung cancer (see Supplementary File S8).

Supplementary File S6. Converting annual probabilities into 3-month probabilities.

3-month probabilities were needed for model. These were calculated by converting annual probabilities into annual rates and then converting annual rates into 3-month probabilities. These conversions were based on these formulas:

$$r = -\frac{\ln(1-p)}{t}$$

$$p = 1 - \exp(-rt)$$

Where r is the rate and p the probability.

So for example, in the first year since a stage I diagnosis, the annual probability of death was 0.088 on average. Therefore, the annual rate was calculated as follows:

$$r = -\frac{\ln(1-0.088)}{1} = 0.092$$

And the 3-month probability was:

$$p = 1 - \exp\left(-\frac{0.092}{4}\right) = 0.023$$

The formulas were taken from [82].

Supplementary File S7. Probabilities of death from lung cancer.

The 3-month probabilities of death from lung cancer during the first five years since diagnosis were adjusted by stage combining these parameters:

- the probabilities of death from lung cancer by age group, sex and year since diagnosis, calculated based on cancers diagnosed from 2010 to 2019 in the Trieste province, reported in the FVG registry [25] (see Figure S2). In particular, based on the probabilities of death from LC at ages 55-69 and 70-84, taken from the FVG registry, we calculated weighted averages for the age groups 66-70, 67-71, 68-72, 69-73. The weights were the number of residents within each sex and age group in the Trieste province in 2019 [69].
- the ratios comparing LC mortality in stage II, III and IV vs. stage I (calculated from [23] and reported in Table S2 in Supplementary File S1).

The stage ratios were applied making sure that the weighted average of the probabilities of death across the different stages was equal to the probability in the FVG registry [25]. The weighted average was calculated using the proportions of people in each lung cancer stage at diagnosis with standard care (calculated from [23] and reported in Table S1 in Supplementary File S1).

Table S13 shows the 3-month probabilities of death from lung cancer. Although Table S13 shows the point estimates, the model values changed based on beta distributions for the annual probabilities of LC death by age group, sex and year since diagnosis, taken from the FVG registry [25], and beta distributions for the annual probabilities of LC death by stage and year since diagnosis, taken from [23].

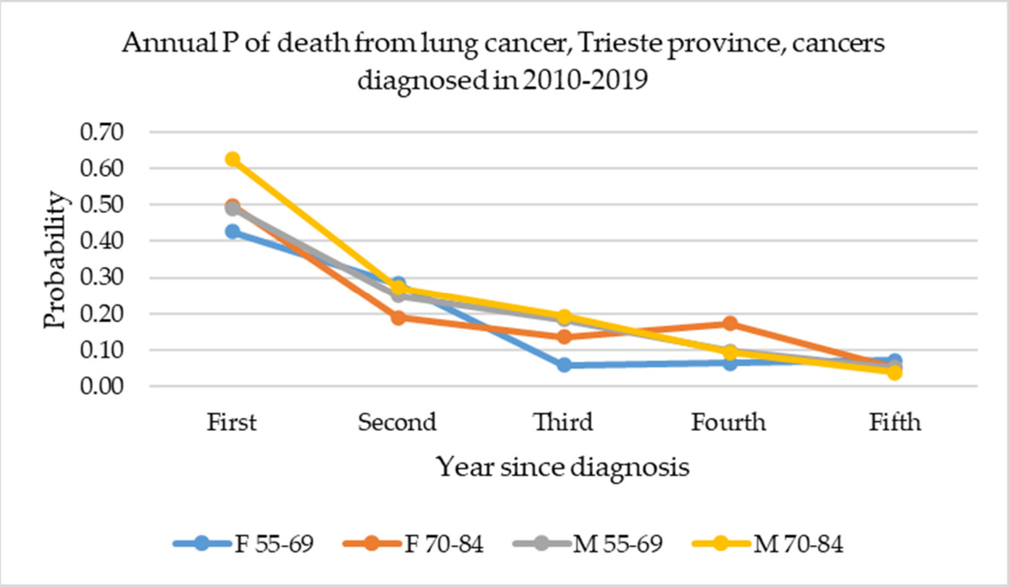

Figure S2. Point estimates of the annual probabilities of death from LC by age group, sex, and year since diagnosis, before adjustment by stage. The sample size for each combination of age group, sex and year since diagnosis ranged from 282 to 2642.

Figure notes. Abbreviations: P: probability.

Table S13. Point estimates of the 3-month probabilities of death from LC after adjusting by stage.

Year since diagnosis	Sex	Stage	Ages					
			From 55-59 to 65-69	66-70	67-71	68-72	69-73	From 70-74 to 99-100
First	Males	I	0.026	0.028	0.030	0.033	0.035	0.037
		II	0.052	0.057	0.061	0.065	0.069	0.074
		III	0.123	0.133	0.143	0.153	0.162	0.173
		IV	0.272	0.294	0.315	0.336	0.357	0.381
	Females	I	0.022	0.023	0.024	0.025	0.026	0.027
		II	0.044	0.046	0.048	0.050	0.052	0.054
		III	0.103	0.108	0.112	0.117	0.121	0.126
		IV	0.227	0.237	0.248	0.257	0.267	0.277
Second	Males	I	0.015	0.016	0.016	0.016	0.017	0.017
		II	0.037	0.038	0.038	0.039	0.040	0.040
		III	0.069	0.070	0.072	0.073	0.074	0.075
		IV	0.106	0.109	0.111	0.113	0.115	0.117
	Females	I	0.018	0.016	0.015	0.014	0.013	0.011
		II	0.042	0.039	0.036	0.033	0.030	0.027
		III	0.079	0.073	0.066	0.061	0.056	0.051
		IV	0.122	0.112	0.103	0.095	0.087	0.078
Third	Males	I	0.021	0.021	0.021	0.022	0.022	0.022
		II	0.032	0.032	0.033	0.033	0.033	0.034
		III	0.045	0.045	0.046	0.046	0.047	0.047
		IV	0.072	0.073	0.074	0.075	0.075	0.076
	Females	I	0.006	0.008	0.010	0.012	0.013	0.015
		II	0.010	0.013	0.015	0.018	0.020	0.023
		III	0.014	0.017	0.021	0.025	0.029	0.032
		IV	0.022	0.028	0.035	0.040	0.046	0.052
Fourth	Males	I	0.011	0.011	0.011	0.011	0.011	0.011
		II	0.022	0.022	0.022	0.022	0.021	0.021
		III	0.030	0.030	0.030	0.029	0.029	0.029
		IV	0.030	0.030	0.029	0.029	0.029	0.029
	Females	I	0.007	0.010	0.012	0.015	0.017	0.020
		II	0.014	0.020	0.025	0.030	0.035	0.040
		III	0.020	0.027	0.034	0.041	0.048	0.055
		IV	0.019	0.027	0.034	0.041	0.047	0.054

Continuation of Table S13. Point estimates of the 3-month probabilities of death from LC after adjusting by stage.

Year since diagnosis	Sex	Stage	Ages					
			From 55-59 to 65-69	66-70	67-71	68-72	69-73	From 70-74 to 99-100
Fifth	Males	I	0.007	0.007	0.006	0.006	0.006	0.005
		II	0.009	0.009	0.008	0.008	0.007	0.007
		III	0.012	0.012	0.011	0.010	0.010	0.009
		IV	0.017	0.016	0.015	0.014	0.014	0.013
	Females	I	0.010	0.009	0.009	0.008	0.008	0.007
		II	0.013	0.012	0.011	0.010	0.010	0.009
		III	0.018	0.017	0.015	0.014	0.014	0.013
		IV	0.024	0.023	0.021	0.020	0.019	0.017

Mortality from LC after the 5th year.

After the 5th year, the 3-month probability of death from lung cancer was 0.013 across all stages. This was based on the 5-year probability of death from lung cancer conditional on having survived for the first 5 years since diagnosis, which was 0.231 [26]: a report by Associazione Italiana di Oncologia Medica (AIOM) and other organizations focused on people who were alive at 5 years since diagnosis, reporting that 72% of them were alive at 10 years [26]. They also mentioned that if >95% had been alive, then the excess risk of death from lung cancer would be negligible compared to the general population of the same age and sex [26]. Therefore, considering that 100%-95.1% is equal to 4.9%, it was assumed that 4.9% had died from other causes, and the 5-year probability of death from lung cancer from year 5 to 10 was 23.1% (100%-72%-4.9%). Note that this 3-month probability of death from lung cancer (0.013) was applied to all stages from the 6th year since diagnosis until death. This was a model simplification.

Supplementary File S8. Mortality from causes other than lung cancer by age and sex.

The annual probability of death from other causes by age and sex was calculated using parameters presented in Table S14. In particular, the parameters included the standardized mortality ratios comparing people formerly exposed to asbestos to the general population in relation to causes other than lung cancer, based on [73].

Table S14. Parameters used to calculate the probability of death from other causes.

Parameter	Mean	Source
Annual probability of death for the general population	Changes by age and sex, see Table S15	[70]
Proportion of deaths due to any cancer, males, general population	0.278	[71], p. 2
Proportion of deaths due to any cancer, females, general population	0.217	[71], p. 2
Proportion of cancer deaths due to LC, males, general population	0.223	[72], p.74
Proportion of cancer deaths due to LC, females, general population	0.138	[72], p.74
Proportion of deaths due to LC, males, general population	0.062	Linked to parameters above
Proportion of deaths due to LC, females, general population	0.030	Linked to parameters above
Male cohort in [73]: Number of deaths from all causes observed among workers formerly exposed to asbestos	31,130	[73]
Male cohort in [73]: Number of deaths from lung cancer observed among workers formerly exposed to asbestos	3535	[73]
Male cohort in [73]: Number of deaths from causes other than lung cancer observed among workers formerly exposed to asbestos	27,595	Linked to parameters above
Male cohort in [73]: Number of deaths from all causes expected if mortality was as in the general population	29,997.53	[73]
Male cohort in [73]: Number of deaths from lung cancer expected if mortality was as in the general population	2760.01	[73]
Male cohort in [73]: Number of deaths from causes other than lung cancer expected if mortality was as in the general population	27,237.52	Linked to parameters above
SMR comparing mortality from causes other than lung cancer in asbestos-exposed vs. general population, males	1.01	Linked to parameters above
Female cohort in [73]: Number of deaths from all causes observed among workers formerly exposed to asbestos	3739	[73]

Parameter	Mean	Source
Female cohort in [73]: Number of deaths from lung cancer observed among workers formerly exposed to asbestos	99	[73]
Female cohort in [73]: Number of deaths from causes other than lung cancer observed among workers formerly exposed to asbestos	3640	Linked to parameters above
Female cohort in [73]: Number of deaths from all causes expected if mortality was as in the general population	3266.54	[73]
Female cohort in [73]: Number of deaths from lung cancer expected if mortality was as in the general population	78.68	[73]
Female cohort in [73]: Number of deaths from causes other than lung cancer expected if mortality was as in the general population	3187.86	Linked to parameters above
SMR comparing mortality not from LC in asbestos-exposed to general population, females	1.14	Linked to parameters above

Table notes. Abbreviations: SMR: standardized mortality ratio.

Table S15 shows the annual probabilities by age and sex. The model used 3-month probabilities, shown in Table S16.

In the model, the weighted average for each age group is assigned based on the model year: for example, the weighted average for the age group 61-65 is applied to the second model year for the age group 60-64 and to the seventh model year for the age group 55-59.

See Table S15 below. Note that the annual P of death for causes other than LC was higher for men than for women when the same age groups were compared. There were these exceptions to this: for those that reached the ages 96-100 / 97-100 / 98-100 / 99-100, mortality was higher for women.

Table S15. Annual P of death from other causes by age

Age	**Annual P of death, all causes, FVG general population, 2022** [70]		**Annual P of LC death, FVG general population, 2022**		**Annual P of death from causes other than LC, FVG general population**		**Annual P of death from causes other than LC, people formerly exposed to asbestos**[1]		**Weights: N of people resident in FVG in 2023** [74]		**Weighted average of the annual P of death for causes other than LC among people formerly exposed to asbestos**		
**Age**	**Men**	**Women**	**Men**	**Women**	**Men**	**Women**	**Men**	**Women**	**N men**	**N women**	**Age group**	**Men**	**Women**
55	0.0039	0.0022	0.0002	0.0001	0.0036	0.0021	0.0037	0.0024	10007	10254	55-59	0.0042	0.0030
56	0.0039	0.0024	0.0002	0.0001	0.0037	0.0024	0.0037	0.0027	10012	10267	56-60	0.0047	0.0033
57	0.0042	0.0028	0.0003	0.0001	0.0039	0.0027	0.0040	0.0031	10150	10253	57-61	0.0053	0.0036
58	0.0047	0.0031	0.0003	0.0001	0.0044	0.0030	0.0045	0.0034	10324	10436	58-62	0.0061	0.0038
59	0.0054	0.0032	0.0003	0.0001	0.0050	0.0031	0.0051	0.0036	9616	10011	59-63	0.0070	0.0042
60	0.0065	0.0034	0.0004	0.0001	0.0061	0.0033	0.0061	0.0038	9140	9425	60-64	0.0079	0.0047
61	0.0074	0.0037	0.0005	0.0001	0.0070	0.0036	0.0071	0.0041	8968	9113	61-65	0.0087	0.0052
62	0.0085	0.0041	0.0005	0.0001	0.0080	0.0039	0.0081	0.0045	8625	8715	62-66	0.0094	0.0059
63	0.0094	0.0047	0.0006	0.0001	0.0089	0.0045	0.0090	0.0052	8339	8749	63-67	0.0101	0.0065
64	0.0102	0.0054	0.0006	0.0002	0.0096	0.0052	0.0097	0.0059	7699	8328	64-68	0.0110	0.0072
65	0.0106	0.0060	0.0007	0.0002	0.0099	0.0058	0.0101	0.0067	7384	8198	65-69	0.0120	0.0078
66	0.0110	0.0065	0.0007	0.0002	0.0104	0.0063	0.0105	0.0072	7440	7915	66-70	0.0134	0.0085
67	0.0122	0.0070	0.0008	0.0002	0.0114	0.0068	0.0116	0.0078	7186	7745	67-71	0.0151	0.0093
68	0.0138	0.0077	0.0009	0.0002	0.0130	0.0075	0.0131	0.0085	6926	7706	68-72	0.0169	0.0103
69	0.0157	0.0083	0.0010	0.0003	0.0147	0.0081	0.0149	0.0092	6806	7649	69-73	0.0189	0.0114
70	0.0183	0.0090	0.0011	0.0003	0.0172	0.0088	0.0174	0.0100	6573	7682	70-74	0.0210	0.0129
71	0.0202	0.0101	0.0013	0.0003	0.0189	0.0098	0.0192	0.0112	6487	7520	71-75	0.0232	0.0146
72	0.0213	0.0112	0.0013	0.0003	0.0200	0.0109	0.0202	0.0124	6844	7927	72-76	0.0259	0.0163
73	0.0238	0.0129	0.0015	0.0004	0.0223	0.0125	0.0226	0.0142	6771	7765	73-77	0.0287	0.0181
74	0.0266	0.0147	0.0016	0.0004	0.0249	0.0143	0.0253	0.0163	7130	8168	74-78	0.0320	0.0204
75	0.0297	0.0165	0.0018	0.0005	0.0279	0.0160	0.0283	0.0182	6852	8205	75-79	0.0357	0.0231
76	0.0344	0.0181	0.0021	0.0005	0.0323	0.0175	0.0327	0.0200	7176	8492	76-80	0.0401	0.0266

Age	Annual P of death, all causes, FVG general population, 2022 [70]		Annual P of LC death, FVG general population, 2022		Annual P of death from causes other than LC, FVG general population		Annual P of death from causes other than LC, people formerly exposed to asbestos[1]		Weights: N of people resident in FVG in 2023 [74]		Weighted average of the annual P of death for causes other than LC among people formerly exposed to asbestos		
	**Men**	**Women**	**Men**	**Women**	**Men**	**Women**	**Men**	**Women**	**N men**	**N women**	**Age group**	**Men**	**Women**
77	0.0388	0.0207	0.0024	0.0006	0.0364	0.0201	0.0369	0.0230	4752	5742	77-81	0.0449	0.0308
78	0.0422	0.0237	0.0026	0.0007	0.0396	0.0230	0.0401	0.0263	5470	6813	78-82	0.0492	0.0347
79	0.0462	0.0271	0.0029	0.0008	0.0433	0.0263	0.0439	0.0300	5244	6583	79-83	0.0539	0.0394
80	0.0523	0.0314	0.0032	0.0009	0.0490	0.0304	0.0497	0.0348	5038	6686	80-84	0.0596	0.0445
81	0.0571	0.0353	0.0035	0.0011	0.0535	0.0343	0.0542	0.0391	4964	6555	81-85	0.0654	0.0500
82	0.0623	0.0390	0.0039	0.0012	0.0584	0.0378	0.0592	0.0432	4954	6719	82-86	0.0729	0.0565
83	0.0680	0.0452	0.0042	0.0014	0.0638	0.0438	0.0646	0.0501	4387	6327	83-87	0.0822	0.0649
84	0.0778	0.0518	0.0048	0.0016	0.0730	0.0502	0.0739	0.0573	3994	5837	84-88	0.0930	0.0746
85	0.0862	0.0581	0.0053	0.0017	0.0809	0.0563	0.0819	0.0643	3390	5074	85-89	0.1070	0.0872
86	0.1032	0.0681	0.0064	0.0020	0.0968	0.0661	0.0981	0.0754	2745	4589	86-90	0.1237	0.1022
87	0.1160	0.0783	0.0072	0.0023	0.1088	0.0759	0.1102	0.0867	2405	4220	87-91	0.1404	0.1183
88	0.1308	0.0914	0.0081	0.0027	0.1227	0.0887	0.1243	0.1012	1875	3709	88-92	0.1597	0.1372
89	0.1576	0.1120	0.0098	0.0034	0.1479	0.1087	0.1498	0.1241	1623	3233	89-93	0.1783	0.1568
90	0.1787	0.1297	0.0111	0.0039	0.1677	0.1258	0.1699	0.1437	1287	2899	90-94	0.1970	0.1751
91	0.1971	0.1447	0.0122	0.0043	0.1848	0.1403	0.1873	0.1602	1055	2476	91-95	0.2174	0.1947
92	0.2168	0.1638	0.0134	0.0049	0.2034	0.1589	0.2061	0.1814	873	2272	92-96	0.2419	0.2186
93	0.2317	0.1816	0.0144	0.0054	0.2174	0.1761	0.2202	0.2011	557	1788	93-97	0.2705	0.2460
94	0.2638	0.2018	0.0164	0.0061	0.2474	0.1957	0.2507	0.2235	452	1373	94-98	0.3010	0.2764
95	0.3125	0.2267	0.0194	0.0068	0.2931	0.2199	0.2969	0.2511	314	1138	95-99	0.3351	0.3092
96	0.3661	0.2691	0.0227	0.0081	0.3434	0.2610	0.3479	0.2980	208	910	96-100	0.4450	0.4624
97	0.3812	0.3034	0.0236	0.0091	0.3576	0.2943	0.3623	0.3361	137	641	97-100	0.4991	0.5403
98	0.3856	0.3375	0.0239	0.0101	0.3617	0.3274	0.3664	0.3738	89	444	98-100	0.5786	0.6426

Age	Annual P of death, all causes, FVG general population, 2022 [70]		Annual P of LC death, FVG general population, 2022		Annual P of death from causes other than LC, FVG general population		Annual P of death from causes other than LC, people formerly exposed to asbestos[1]		Weights: N of people resident in FVG in 2023 [74]		Weighted average of the annual P of death for causes other than LC among people formerly exposed to asbestos		
	**Men**	**Women**	**Men**	**Women**	**Men**	**Women**	**Men**	**Women**	**N men**	**N women**	**Age group**	**Men**	**Women**
99	0.3956	0.3693	0.0245	0.0111	0.3710	0.3582	0.3759	0.4090	69	303	99-100	0.7071	0.7855
100	1	1	NA	NA	NA	NA	1	1	78	532	100	NA	NA

Table notes. Abbreviations: FVG: Friuli Venezia Giulia; LC: lung cancer; P: probability.

[1] The annual P of death from causes other than LC in the general population was multiplied by a SMR (standardized mortality ratio) comparing people formerly exposed to asbestos vs. the general population (see table S14).

Annual P of death was taken from [70] for ages 55-99. For age 100, it was assumed that P=1 (model simplification).

Table S16. 3-month probabilities of death from causes other than lung cancer.

Model year	Males 55-59	Males 60-64	Males 65-69	Males 70-74	Males 75-79	Males 80	Females 55-59	Females 60-64	Females 65-69	Females 70-74	Females 75-79	Females 80
1	0.0010	0.0020	0.0030	0.0053	0.0090	0.0127	0.0008	0.0012	0.0020	0.0032	0.0058	0.0088
2	0.0012	0.0022	0.0034	0.0058	0.0102	0.0138	0.0008	0.0013	0.0021	0.0037	0.0067	0.0099
3	0.0013	0.0024	0.0038	0.0065	0.0114	0.0151	0.0009	0.0015	0.0023	0.0041	0.0078	0.0110
4	0.0015	0.0025	0.0043	0.0072	0.0125	0.0166	0.0010	0.0016	0.0026	0.0046	0.0088	0.0128
5	0.0018	0.0028	0.0048	0.0081	0.0138	0.0190	0.0011	0.0018	0.0029	0.0051	0.0100	0.0146
6	0.0020	0.0030	0.0053	0.0090	0.0152	0.0211	0.0012	0.0020	0.0032	0.0058	0.0113	0.0165
7	0.0022	0.0034	0.0058	0.0102	0.0168	0.0255	0.0013	0.0021	0.0037	0.0067	0.0127	0.0194
8	0.0024	0.0038	0.0065	0.0114	0.0187	0.0288	0.0015	0.0023	0.0041	0.0078	0.0144	0.0224
9	0.0025	0.0043	0.0072	0.0125	0.0212	0.0326	0.0016	0.0026	0.0046	0.0088	0.0166	0.0263
10	0.0028	0.0048	0.0081	0.0138	0.0241	0.0398	0.0018	0.0029	0.0051	0.0100	0.0192	0.0326
11	0.0030	0.0053	0.0090	0.0152	0.0279	0.0455	0.0020	0.0032	0.0058	0.0113	0.0225	0.0380
12	0.0034	0.0058	0.0102	0.0168	0.0325	0.0505	0.0021	0.0037	0.0067	0.0127	0.0266	0.0427
13	0.0038	0.0065	0.0114	0.0187	0.0371	0.0561	0.0023	0.0041	0.0078	0.0144	0.0310	0.0488
14	0.0043	0.0072	0.0125	0.0212	0.0426	0.0603	0.0026	0.0046	0.0088	0.0166	0.0362	0.0546
15	0.0043	0.0072	0.0125	0.0212	0.0426	0.0696	0.0026	0.0046	0.0088	0.0166	0.0362	0.0613
16	0.0053	0.0090	0.0152	0.0279	0.0534	0.0843	0.0032	0.0058	0.0113	0.0225	0.0470	0.0697
17	0.0058	0.0102	0.0168	0.0325	0.0594	0.1014	0.0037	0.0067	0.0127	0.0266	0.0527	0.0847
18	0.0065	0.0114	0.0187	0.0371	0.0669	0.1064	0.0041	0.0078	0.0144	0.0310	0.0598	0.0973
19	0.0072	0.0125	0.0212	0.0426	0.0758	0.1078	0.0046	0.0088	0.0166	0.0362	0.0681	0.1104
20	0.0081	0.0138	0.0241	0.0479	0.0856	0.1112	0.0051	0.0100	0.0192	0.0417	0.0777	0.1232
21	0.0090	0.0152	0.0279	0.0534	0.0970	1.0000	0.0058	0.0113	0.0225	0.0470	0.0883	1.0000
22	0.0102	0.0168	0.0325	0.0594	0.1369	1.0000	0.0067	0.0127	0.0266	0.0527	0.1437	1.0000
23	0.0114	0.0187	0.0371	0.0669	0.1587	1.0000	0.0078	0.0144	0.0310	0.0598	0.1766	1.0000
24	0.0125	0.0212	0.0426	0.0758	0.1943	1.0000	0.0088	0.0166	0.0362	0.0681	0.2268	1.0000
25	0.0138	0.0241	0.0479	0.0856	0.2643	1.0000	0.0100	0.0192	0.0417	0.0777	0.3195	1.0000
26	0.0152	0.0279	0.0534	0.0970	1.0000	1.0000	0.0113	0.0225	0.0470	0.0883	1.0000	1.0000
27	0.0168	0.0325	0.0594	0.1369	1.0000	1.0000	0.0127	0.0266	0.0527	0.1437	1.0000	1.0000
28	0.0187	0.0371	0.0669	0.1587	1.0000	1.0000	0.0144	0.0310	0.0598	0.1766	1.0000	1.0000
29	0.0212	0.0426	0.0758	0.1943	1.0000	1.0000	0.0166	0.0362	0.0681	0.2268	1.0000	1.0000
30	0.0241	0.0479	0.0856	0.2643	1.0000	1.0000	0.0192	0.0417	0.0777	0.3195	1.0000	1.0000
31	0.0279	0.0534	0.0970	1.0000	1.0000	1.0000	0.0225	0.0470	0.0883	1.0000	1.0000	1.0000
32	0.0325	0.0594	0.1369	1.0000	1.0000	1.0000	0.0266	0.0527	0.1437	1.0000	1.0000	1.0000
33	0.0371	0.0669	0.1587	1.0000	1.0000	1.0000	0.0310	0.0598	0.1766	1.0000	1.0000	1.0000
34	0.0426	0.0758	0.1943	1.0000	1.0000	1.0000	0.0362	0.0681	0.2268	1.0000	1.0000	1.0000
35	0.0479	0.0856	0.2643	1.0000	1.0000	1.0000	0.0417	0.0777	0.3195	1.0000	1.0000	1.0000
36	0.0534	0.0970	1.0000	1.0000	1.0000	1.0000	0.0470	0.0883	1.0000	1.0000	1.0000	1.0000
37	0.0594	0.1369	1.0000	1.0000	1.0000	1.0000	0.0527	0.1437	1.0000	1.0000	1.0000	1.0000

Model year	Males 55-59	Males 60-64	Males 65-69	Males 70-74	Males 75-79	Males 80	Females 55-59	Females 60-64	Femal es 65-69	Femal es 70-74	Female s 75-79	Femal es 80
38	0.0669	0.1587	1.0000	1.0000	1.0000	1.0000	0.0598	0.1766	1.0000	1.0000	1.0000	1.0000
39	0.0758	0.1943	1.0000	1.0000	1.0000	1.0000	0.0681	0.2268	1.0000	1.0000	1.0000	1.0000
40	0.0856	0.2643	1.0000	1.0000	1.0000	1.0000	0.0777	0.3195	1.0000	1.0000	1.0000	1.0000
41	0.0970	1.0000	1.0000	1.0000	1.0000	1.0000	0.0883	1.0000	1.0000	1.0000	1.0000	1.0000
42	0.1369	1.0000	1.0000	1.0000	1.0000	1.0000	0.1437	1.0000	1.0000	1.0000	1.0000	1.0000
43	0.1587	1.0000	1.0000	1.0000	1.0000	1.0000	0.1766	1.0000	1.0000	1.0000	1.0000	1.0000
44	0.1943	1.0000	1.0000	1.0000	1.0000	1.0000	0.2268	1.0000	1.0000	1.0000	1.0000	1.0000
45	0.2643	1.0000	1.0000	1.0000	1.0000	1.0000	0.3195	1.0000	1.0000	1.0000	1.0000	1.0000
46	1.0000	1.0000	1.0000	1.0000	1.0000	1.0000	1.0000	1.0000	1.0000	1.0000	1.0000	1.0000

Supplementary File S9. Calculation of utilities

Table S17. Utilities and related parameters

Parameter	Mean	95% CI LL	95% CI UL	Distribution	Distribution parameters: Alpha / mean	Distribution parameters: Beta / SE	Source
**Utilities from Italy: general population**							
Utility of general population aged 55+	0.91	0.896	0.924	Normal	0.91	0.007	[27]
Utility, general population, males aged 18 and over	0.94	0.932	0.948	Normal	0.94	0.004	[27]
Utility, general population, females aged 18 and over	0.92	0.910	0.930	Normal	0.92	0.005	[27]
Utility, aged 18 and over	0.93	0.924	0.936	Normal	0.93	0.003	[27]
Ratio between utility for men aged 18 and over and for all aged 18 and over	1.01	Linked to parameters above					[27]
Ratio between utility for women aged 18 and over and for all aged 18 and over	0.99	Linked to parameters above					[27]
Utility, men aged 55+	0.92	Linked to parameters above					Par above
Utility, women aged 55+	0.90	Linked to parameters above					Par above
**Utilities from England (used to calculate ratios comparing current and former smoking vs. the general population)**							
Utility, general population aged 16+	0.851	0.847	0.855	Normal	0.851	0.00002	[28]
Utility, former smoking, aged 16+	0.818	0.810	0.826	Normal	0.818	0.00007	[28]
Smoking 10 to <20 per day, age 16+ [1]	0.828	0.813	0.843	Normal	0.828	0.00022	[28]
**Calculating ratios based on English data**							
Utilities: ratio comparing former smoking vs. general population	0.961	Linked to parameters above					[28]
Utilities: ratio comparing 10 to <20 cigarettes per day vs. general population	0.973	Linked to parameters above					[28]
**Combining the ratios from England with Italian data to estimate utilities for Italian people who smoke[2]**							
Utilities, former smoking, men 55+	0.884	Linked to parameters above					Par above
Utilities, former smoking, women 55+	0.865	Linked to parameters above					Par above
Utilities, former smoking, both sexes[3]	0.875	Linked to parameters above					Par above
Utilities, smoking 10 to <20 per day, men 55+	0.895	Linked to parameters above					Par above
Utilities, smoking 10 to <20 per day, women 55+	0.876	Linked to parameters above					Par above
Utilities, smoking 10 to <20 per day, both sexes[3]	0.885	Linked to parameters above					Par above
Weights for weighted average: proportion of people who formerly	0.77	0.70	0.83	Beta	527	160	[22]

Parameter	Mean	95% CI		Distribution	Distribution parameters		Source
		LL	UL		Alpha / mean	Beta / SE	
smoked among people who ever smoked in [22]							
Weights for weighted average: proportion of people who currently smoke among people who ever smoked in [22]	0.23	Linked to parameters above					[22]
Utility: weighted average, current and former smoking, men 55+	0.887	Linked to parameters above					Par above
Utility: weighted average, current and former smoking, women 55+	0.868	Linked to parameters above					Par above
Utility: weighted average, current and former smoking, both sexes 55+[3]	0.877	Linked to parameters above					Par above
**Utilities by age group and sex for people smoking moderately or formerly smoking in England (used to calculate ratios comparing different age groups)**							
Utility for males aged 55-64, moderate smoking, England	0.782	0.768	0.795	Normal	0.782	0.007	[29]
Utility for males aged 55-64, formerly smoking, England	0.802	0.792	0.812	Normal	0.802	0.005	[29]
Weighted average, males 55-64, currently & formerly smoking, England	0.797	Linked to parameters above					
Utility for males aged 65-74, moderate smoking, England	0.758	0.742	0.773	Normal	0.758	0.008	[29]
Utility for males aged 65-74, formerly smoking, England	0.780	0.769	0.792	Normal	0.780	0.006	[29]
Weighted average, males 65-74, currently & formerly smoking, England	0.775	Linked to parameters above					
Utility for males aged 75+, moderate smoking, England	0.711	0.695	0.727	Normal	0.711	0.008	[29]
Utility for males aged 75+, formerly smoking, England	0.736	0.724	0.747	Normal	0.736	0.006	[29]
Weighted average, males 75+, currently & formerly smoking, England	0.730	Linked to parameters above					
Ratio: utility for males aged 65-74 / utility for males aged 55-64	0.972	Linked to parameters above					Par above
Ratio: utility for males aged 75+ / utility for males aged 55-64	0.916	Linked to parameters above					Par above
Utility for females aged 55-64, moderate smoking, England	0.765	0.751	0.778	Normal	0.765	0.007	[29]
Utility for females aged 55-64, formerly smoking, England	0.783	0.773	0.793	Normal	0.783	0.005	[29]
Weighted average, females, aged 55-64, formerly and currently smoking	0.779	Parameters above					
Utility for females aged 65-74, moderate smoking, England	0.752	0.737	0.767	Normal	0.752	0.008	[29]

Parameter	Mean	95% CI		Distribution	Distribution parameters		Source
		LL	UL		Alpha / mean	Beta / SE	
Utility for females aged 65-74, formerly smoking, England	0.771	0.760	0.782	Normal	0.771	0.006	[29]
Weighted average, females, aged 65-74, formerly and currently smoking	0.766	Parameters above					
Utility for females aged 75+, moderate smoking, England	0.678	0.661	0.695	Normal	0.678	0.009	[29]
Utility for females aged 75+, formerly smoking, England	0.699	0.686	0.712	Normal	0.699	0.007	[29]
Weighted average, females, aged 75+, formerly and currently smoking	0.694	Parameters above					
Ratio: utility for females aged 65-74 / utility for females aged 55-64	0.985	Linked to parameters above					Par above
Ratio: utility for females aged 75+ / utility for females aged 55-64	0.891	Linked to parameters above					Par above
**Utilities for people without lung cancer by age group and sex (applied in the model): see Table S3 in Supplementary File S1. They were calculated based on the parameters above and the proportions in each age group from [21].**							
**Utilities from Italy: people with lung cancer**							
Utility with lung cancer, all stages, males and females, Italy	0.850	0.829	0.871	Normal	0.85	0.011	[30]
**Combining the utilities for Italian people who smoke with the utilities for people with lung cancer**							
Ratio between the utilities for lung cancer vs. the utilities for people currently or formerly smoking (both sexes, all age groups 55+)	0.969	Linked to parameters above					Par above
Utility for lung cancer, all stages, males aged 55-64, Italy	0.896	Linked to the ratio above and the utilities for an absence of lung cancer in this subgroup					Par above
Utility for lung cancer, all stages, males aged 65-74, Italy	0.871	Same as row above					Par above
Utility for lung cancer, all stages, males aged 75-80, Italy	0.821	Same as row above					Par above
Utility for lung cancer, all stages, females aged 55-64, Italy	0.882	Same as row above					Par above
Utility for lung cancer, all stages, females aged 65-74, Italy	0.869	Same as row above					Par above
Utility for lung cancer, all stages, females aged 75-80, Italy	0.786	Same as row above					Par above
**Utilities from the United States (used to calculated multipliers for stages II, III, IV vs. I)**							
Utility for stage I lung cancer	0.810	0.798	0.822	Normal	0.810	0.006	[31]
Utility for stage II lung cancer	0.770	0.748	0.792	Normal	0.770	0.011	[31]
Utility for stage III lung cancer	0.770	0.756	0.784	Normal	0.770	0.007	[31]
Utility for stage IV lung cancer	0.760	0.745	0.775	Normal	0.760	0.008	[31]

Parameter	Mean	95% CI		Distribution	Distribution parameters		Source
		LL	UL		Alpha / mean	Beta / SE	
Utilities ratio: stage II vs. I	0.951	Linked to parameters above					Par above
Utilities ratio: stage III vs. I	0.951	Linked to parameters above					Par above
Utilities ratio: stage IV vs. I	0.938	Linked to parameters above					Par above
**Utilities for each LC stage, sex and age group: see Table S3 in Supplementary File S1 for the utilities, which were calculated using the parameters above and the stage distribution at screening.** [4]							

Table notes. Abbreviations: CI: confidence interval; LL: lower limit; NR: not reported; QALY: quality-adjusted life year; Par: parameter(s); UL: upper limit. Colour legend: light blue: males; amber: females; white: not sex-specific. Distribution parameters: alpha and beta for beta distribution, mean and SE for normal distribution, shape and scale for gamma distribution.

[1] The study separately reported utilities for people smoking less than 10 cigarettes per day, 10 to <20 and at least 20 cigarettes per day. For simplicity, the value in the middle (from 10 to <20) was selected.

[2] The multipliers based on English data were applied to the utilities for the Italian general population taking into account the proportions of people who formerly smoked and who currently smoke in [22].

[3] Only used to compare utilities with and without lung cancer.

[4] Using the stage distribution at screening, reported in Table S1 in Supplementary File S1, the utilities ratios comparing different stages were combined with the utilities for lung cancer in each subgroup to calculate the utilities for each stage, age group and sex, presented in Table S3 in Supplementary File S1.

Supplementary File S10. Some simplifications made in the diagnostic pathways

Various simplifications were made in the diagnostic pathways represented in Figure 2, which is used to calculate the detection costs and false positive costs in the model. Here we provide a summary of these simplifications: firstly, the ASUGI working group considered that a first health care visit in the standard care arm could be with a GP, with an occupational health specialist, with another specialist or in A&E. However, in the absence of data on the different probabilities corresponding to each of these settings, the inflated-to-2023 cost of a "first visit" ("Prima visita escluso le prime visite specificatamente codificate", Codice: 89.7 [33]) of EUR 31.51 was applied. This was a model simplification.
Moreover, the model simplified the diagnostic pathways in Figure 2 by not considering that in some cases a needle biopsy is done instead of endoscopic investigations, and in some cases a brain CT is done instead of a brain MRI. These simplifications were done due to time constraints combined with the complexity of using local data to calculate the probability of having a CT-guided needle biopsy instead of endoscopic investigations such as bronchoscopy, or the probability of having a combination of both, and the complexity of using local data to calculate the probability of having a brain CT instead of a brain MRI.
Furthermore, for simplicity, under the additional investigations, only the pneumology visit was considered, although in practice, in some cases, a thoracic surgery visit is done instead of a pneumology visit.
Additionally, the pathways in Figure 2 did not include diagnostic complications such as pneumothorax or bleeding.

For the diagnostic and treatment costs applied to people with lung cancer, Figure 2 was not used: these costs were taken from a previous study [13]. It was assumed that these costs would include any diagnostic complications such as pneumothorax or bleeding.

Supplementary File S11. Calculation of probabilities relating to the false positives.

Intervention arm

Across all ages, the probability of a false positive with an LDCT scan was 18%. This was based on an estimate by the International Agency for Research on Cancer for people screened annually for 3 years with LDCT [37]. The agency calculated that if 1000 eligible individuals were screened 3 times with LDCT, 180 would have an extra scan despite not having lung cancer.

To calculate the ratio comparing people aged <65 and people aged >= 65 in relation to the probability of a false positive with LDCT, we used the numbers reported for the baseline screening round in [38]: for those aged <65, there were 4796 false positive re-sults among 19,306 people screened. For those aged >=65, there were 2125 false positive results among 7003 people screened. Therefore, the point estimate of the ratio comparing the older to the younger age group was 1.221 (30.3%/24.8%).

We were unable to make adjustments for each smaller age group in the model (for example, we applied the same probability of a false positive from LDCT to the age groups 70-74 and 75-79). We made sure that the weighted average of the probabilities of LDCT false positives across all age groups was 18%. The weights for the weighted av-erage were the proportions of people aged <65 and >=65 in [21].

Standard care arm

In the standard care arm, the probability of a false positive with an X-ray was 8.6% on average. This was calculated based on the NLST trial [39]: in the baseline round of chest radiography, 26,035 X-rays were done, and 2251 gave a positive result with no LC confirmed [39].

Intervention arm: probability of HRCT after a false positive

There was no information available on the probability of a highly suspicious LDCT result among false positives, which would skip the HRCT ahead of additional investigations. For simplicity, it was assumed that all false positive LDCT results underwent an HRCT ahead of additional investigations.

Intervention arm: probability of additional investigations (including endoscopic investigations, PET-TC, thoracic-abdominal TC, brain MRI and respiratory function tests) after a false positive

In the intervention arm, the probability of additional investigations after a false positive LDCT was 7.2% on average. This was based on the International Agency for Research on Cancer for people screened annually for 3 years with LDCT [37], which calculated that if 180 screening participants had an extra scan despite not having lung cancer, 13 of them would have an invasive procedure.

Standard care arm: probability of additional investigations (including endoscopic investigations, PET-TC, thoracic-abdominal TC, brain MRI and respiratory function tests) after a false positive

In the standard care arm, the probability of additional investigations if there was a false positive X-ray was 1.5%. This was calculated based on the NLST trial [39]: over three rounds of chest radiography, there were 4674 positive X-rays with no LC confirmed, and 70 of them underwent either a bronchoscopy or a needle biopsy [39].

Intervention arm: probability of surgery in a false positive

In the intervention arm, the probability of surgery if there was a false positive LDCT was 0.8% on average. This was based on the UKLS trial, where 495 people with no lung cancer were either recalled for a repeat scan at 3 months or immediately referred to the MDT, and of these, 4 people had surgery despite not having lung cancer (Figure 14 in [77] (p. 42) presented a flowchart where surgery resulted in either "Not cancer" or in "Pathology confirmed").

Standard care arm: probability of surgery in a false positive.

In the standard care arm, the probability of surgery after a false positive X-ray was 1% on average. This was calculated based on the NLST trial [39]. Over the three rounds of chest radiography, there were 4674

positive X-rays with no LC confirmed, and 45 of them underwent thoracotomy, thoracoscopy or mediastinoscopy [39].

Supplementary File S12. Costs before inflation, with cost code in official documentation, if applicable.

Refer to Supplementary File S1 for the inflated costs.

Table S18. Initial screening and passive surveillance costs (detection costs).

**Parameter**	**Cost in EUR**	**Reference**	**Name and code in Nomenclatore Tariffario FVG 2019** [33]
Admin & operating costs of screening per participant in (assumed)[1] 2019	17.0	[13]	NA
Cost of initial visit to discuss risks and benefits in 2019	29.0	[33]	Prima visita escluso le prime visite specificatamente codificate. Codice: 89.7
Cost of LDCT scan in 2019	119.1	[33]	TC del torace. Codice: 87.41
Cost of a control visit, or of an initial visit due to LC symptoms	29.0	[33]	Prima visita escluso le prime visite specificatamente codificate. Codice: 89.7
Cost of X-Ray in 2019	25.1	[33]	Radiografia del torace di routine, NAS. Codice: 87.44.1.
Cost of global spirometry in 2019	47.4	[33]	Spirometria globale. Codice: 89.37.2.
Cost of DLCO test in 2019	29.6	[33]	Diffusione alveolo-capillare del CO. Codice: 89.38.3

Table notes. Abbreviations: DLCO: diffusing capacity of the lungs for carbon monoxide; LC: lung cancer; LDCT: low-dose computed tomography.

Only the point estimates are included in the table. See the inflated costs in Supplementary File S1 for the cost distributions.

[1] The reference year for costs was not reported in [13]. Considering that the paper was received in October 2019 and published in 2020, it was assumed that costs referred to 2019 for the purpose of inflating them to 2023.

Table S19. Diagnostic and treatment costs for people with lung cancer

**Parameter**	**Cost in EUR**	**Source**
Cost of workup and treatment, stage IA, in (assumed)[1] 2019	28,022	[13]
Cost of workup and treatment, stage IB, in (assumed)[1] 2019	26,772	[13]
Cost of workup and treatment, stage II, in (assumed)[1] 2019	22,776	[13]
Cost of workup and treatment, stage III, in (assumed)[1] 2019	33,343	[13]
Cost of workup and treatment, stage IV, in (assumed)[1] 2019	39,064	[13]

Table notes. Only the point estimates are included in the table. See the inflated costs in Supplementary File S1 for the cost distributions.

[1]The reference year for costs was not reported in [13]. Considering that the paper was received in October 2019 and published in 2020, it was assumed that costs referred to 2019 for the purpose of inflating them to 2023.

Table S20. Cost of the false positives.

Parameter	Mean	95% CI		Distribution	Distribution parameters		Source	Name and code in official sources
		LL	UL		Mean / alpha / shape	SD / beta / scale		
Cost of HRCT without contrast agent in 2019	119.1	NR	NR	NA. 95% CI and distribution calculated for inflated cost			[33]	TC del torace. Codice: 87.41
**Cost of additional investigations after positive HRCT or highly suspicious LDCT**								
Pneumology visit in 2019	39.0	31.7	47.0	Gamma	100	0.39	[33]	Prima visita pneumologica. Codice: 89.7B.9
Body spirometry in 2019	29.6	24.1	35.7	Gamma	100	0.30		Bodyspirometria. Codice: 89.38.2
DLCO test in 2019	29.6	24.1	35.7	Gamma	100	0.30		Test DLCO. Codice: 89.38.3
Walking test in 2019	23.7	19.3	28.6	Gamma	100	0.24		Walking test. Codice: 89.44.2
Bronchoscopy with biopsy in 2019	164.9	134.2	198.8	Gamma	100	1.65		Broncoscopia con biopsia. Codice: 33.24.1
Test for carcinoembryonic antigen in 2019	10.9	8.9	13.1	Gamma	100	0.11		CEA (antigene carcino embrionario). Codice: 90.56.3
PET-CT in 2019	1032.1	839.8	1244.0	Gamma	100	10.32		PET con correlazione TAC corporea senza estremità. Codice: 92.19.8
Thoracic CT in 2019	188.2	153.1	226.8	Gamma	100	1.88		TAC torace. Codice: 87.41.1
Abdominal CT in 2019	248.9	202.5	300.0	Gamma	100	2.49		TAC addome. Codice: 88.01.6.
Brain MRI in 2019	356.8	290.3	430.0	Gamma	100	3.57		RMN encefalo. Codice: 88.91.2
Tot cost of additional investigations (after positive HRCT or highly suspicious LDCT) in 2019	2123.7	NA, sum of parameters above						NA
**Costs of surgery**								
Cost of surgery (assumes admission for >1 day in type A hospital), in 2019	10,200.0	NR	NR	NA. 95% CI and distribution calculated for inflated cost			[34]	Interventi maggiori sul torace, fascia A, DRG 75, MDC 4, Tipo C.

Table notes. Abbreviations: CI: confidence interval; CT: computed tomography; DLCO: diffusing capacity of the lungs for carbon monoxide; HRCT: high resolution computed tomography; LDCT: low-dose computed tomography; LL: lower limit; MRI: magnetic resonance imaging; NA: not applicable; NR: not reported; PET: positron emission tomography; SD: standard deviation; UL: upper limit.

Supplementary File S13. Proportions in the cost-effectiveness acceptability curve.

The table below shows the probabilities of cost-effectiveness corresponding to different thresholds in Figure 3b.

Table S21. Probabilities of cost-effectiveness relative to different thresholds.

ICER threshold	Probability that intervention is cost-effective	Probability that standard care is cost-effective
0	0.0920	0.9080
1000	0.1925	0.8075
2000	0.3250	0.6750
3000	0.4605	0.5395
4000	0.5830	0.4170
5000	0.6795	0.3205
6000	0.7480	0.2520
7000	0.8130	0.1870
8000	0.8560	0.1440
9000	0.8890	0.1110
10000	0.9170	0.0830
11000	0.9300	0.0700
12000	0.9460	0.0540
13000	0.9570	0.0430
14000	0.9680	0.0320
15000	0.9765	0.0235
16000	0.9820	0.0180
17000	0.9830	0.0170
18000	0.9870	0.0130
19000	0.9890	0.0110
20000	0.9895	0.0105
21000	0.9895	0.0105
22000	0.9925	0.0075
23000	0.9925	0.0075
24000	0.9940	0.0060
25000	0.9950	0.0050
26000	0.9955	0.0045
27000	0.9965	0.0035
28000	0.9970	0.0030
29000	0.9970	0.0030
30000	0.9970	0.0030

ICER threshold	Probability that intervention is cost-effective	Probability that standard care is cost-effective
31000	0.9975	0.0025
32000	0.9980	0.0020
33000	0.9980	0.0020
34000	0.9980	0.0020
35000	0.9980	0.0020
36000	0.9980	0.0020
37000	0.9985	0.0015
38000	0.9990	0.0010
39000	0.9990	0.0010
40000	0.9990	0.0010
41000	0.9990	0.0010
42000	0.9990	0.0010
43000	0.9990	0.0010
44000	0.9995	0.0005
45000	0.9995	0.0005
46000	0.9995	0.0005
47000	0.9995	0.0005
48000	0.9995	0.0005
49000	0.9995	0.0005
50000	0.9995	0.0005

Table notes. ICER: incremental cost-effectiveness ratio, calculated by dividing the incremental cost by the number of QALY gained. The probabilities for each threshold sum up to 1 (due to rounding, this is not apparent in some rows of the table above).

Supplementary File S14. Details on the results for each subgroup.

For each subgroup, on average, there was an increase in total costs with the intervention (Table S22 and Table S23), although for the subgroups aged 65+ there was a probability above 5% that the intervention could be cost-saving, for both males and females. This probability ranged between 6.2% for males aged 80 to 20.9% for females aged 75-79.

Table S22. Base case analysis, incremental results for life years, QALYs and total cost, by age subgroup, males. Mean and 95% CI.

Subgroup	Estimate	Life years	QALYs	Total cost	ICER: incremental cost (EUR) per life year saved	ICER: incremental cost (EUR) per additional QALY
Males 55-59	Mean	0.023	0.019	175	7657	8995
	2.5th p	0.007	0.005	95	NA	NA
	97.5th p	0.053	0.046	228	NA	NA
Males 60-64	Mean	0.028	0.024	159	5628	6646
	2.5th p	0.009	0.006	49	NA	NA
	97.5th p	0.065	0.056	223	NA	NA
Males 65-69	Mean	0.049	0.042	122	2488	2943
	2.5th p	0.016	0.012	-49	NA	NA
	97.5th p	0.106	0.092	222	NA	NA
Males 70-74	Mean	0.058	0.048	88	1500	1820
	2.5th p	0.020	0.015	-143	NA	NA
	97.5th p	0.122	0.102	214	NA	NA
Males 75-79	Mean	0.053	0.043	84	1588	1943
	2.5th p	0.018	0.014	-154	NA	NA
	97.5th p	0.111	0.093	217	NA	NA
Males 80	Mean	0.034	0.027	129	3794	4733
	2.5th p	0.011	0.008	-58	NA	NA
	97.5th p	0.072	0.060	235	NA	NA

Table notes. Abbreviations: CI: credible interval; ICER: incremental cost-effectiveness ratio; QALYs: quality-adjusted life years; p: percentile; PSA: probabilistic sensitivity analysis.
Colour legend: light green = intervention more effective than comparator; amber = intervention more expensive than comparator; light blue = intervention cheaper than comparator.
Results are per person participating in the screening programme and refer to a lifetime horizon.

Table S23. Base case analysis, main outcomes, incremental results by age subgroup, females. Mean and 95% CI.

Subgroup	Estimate	Life years	QALYs	Total cost	ICER: incremental cost (EUR) per life year saved	ICER: incremental cost (EUR) per additional QALY
Females 55-59	Mean	0.024	0.020	175	7358	8718
	2.5th p	0.007	0.005	95	NA	NA
	97.5th p	0.054	0.047	228	NA	NA
Females 60-64	Mean	0.030	0.025	158	5283	6293
	2.5th p	0.009	0.007	46	NA	NA
	97.5th p	0.069	0.059	222	NA	NA
Females 65-69	Mean	0.052	0.043	119	2295	2762
	2.5th p	0.017	0.013	-55	NA	NA
	97.5th p	0.111	0.095	221	NA	NA
Females 70-74	Mean	0.060	0.048	80	1336	1668
	2.5th p	0.020	0.015	-160	NA	NA
	97.5th p	0.125	0.102	208	NA	NA
Females 75-79	Mean	0.055	0.043	70	1280	1617
	2.5th p	0.018	0.014	-182	NA	NA
	97.5th p	0.115	0.092	211	NA	NA
Females 80	Mean	0.036	0.028	117	3283	4223
	2.5th p	0.011	0.008	-86	NA	NA
	97.5th p	0.074	0.060	226	NA	NA

Table notes. Abbreviations: CI: credible interval; ICER: incremental cost-effectiveness ratio; QALYs: quality-adjusted life years; p: percentile; PSA: probabilistic sensitivity analysis.

Colour legend: light green = intervention more effective than comparator; amber = intervention more expensive than comparator; light blue = intervention cheaper than comparator.

Results are per person participating in the screening programme and refer to a lifetime horizon.

Lung cancer deaths prevented, subgroup results.

Table S24 shows the number of deaths from lung cancer or from other causes by the end of the model.

Table S24. Base case analysis, average number of deaths from lung cancer and from other causes by the end of the model. Numbers per 10,000 people.

Subgroup	Number of deaths from LC per 10,000 people			Number of deaths from other causes per 10,000 people		
	Intervention	Standard care	Incremental	Intervention	Standard care	Incremental
WA	154.2	193.0	-38.7	9845.8	9807.0	38.7
Males 55-59	66.5	74.5	-8.0	9933.5	9925.5	8.0
Males 60-64	84.0	96.9	-13.0	9916.0	9903.1	13.0
Males 65-69	142.5	172.5	-29.9	9857.5	9827.5	29.9
Males 70-74	186.3	234.7	-48.4	9813.7	9765.3	48.4
Males 75-79	189.3	250.5	-61.2	9810.7	9749.5	61.2
Males 80	135.8	185.7	-49.9	9864.2	9814.3	49.9
Females 55-59	67.6	74.4	-6.8	9932.4	9925.6	6.8
Females 60-64	85.6	96.9	-11.4	9914.4	9903.1	11.4
Females 65-69	144.9	171.5	-26.5	9855.1	9828.5	26.5
Females 70-74	189.3	231.4	-42.1	9810.7	9768.6	42.1
Females 75-79	189.5	244.7	-55.2	9810.5	9755.3	55.2
Females 80	134.1	179.9	-45.8	9865.9	9820.1	45.8

Table notes. Abbreviations: INCR: incremental; INT: intervention; SC: standard care; WA: weighted average. "Incremental" refers to the difference between intervention and standard care.

Overdiagnosis by subgroup

Table S25 shows that overdiagnosis increased by age. This is because mortality from other causes increased by age. Overdiagnosis was higher for males because they had a higher 3-month probability of death from other causes compared to females of the same age.

Note that the proportion diagnosed with the intervention was not exactly what one could expect based on the point estimate of screen-detectable prevalence. This is due to probabilistic sampling from the prevalence parameter distribution.

Table S25. Base case analysis, average number of diagnosed lung cancers per 10,000 people with intervention and standard care, and overdiagnosis estimates.

Subgroup	Intervention	Standard care	Overdiagnosis
Weighted average	251.8	246.8	5.0
Males 55-59	86.2	85.8	0.3
Males 60-64	116.8	115.9	0.9
Males 65-69	215.8	213.4	2.4
Males 70-74	299.7	293.9	5.9
Males 75-79	341.1	329.8	11.4
Males 80	265.4	253.2	12.2
Females 55-59	86.2	85.9	0.2
Females 60-64	116.8	116.3	0.5
Females 65-69	215.8	214.2	1.6
Females 70-74	299.7	296.1	3.6
Females 75-79	341.1	333.7	7.4
Females 80	265.4	256.8	8.6

Table notes. Overdiagnosis is the difference between the proportion of people diagnosed with lung cancer in the intervention and in the standard care arm. It corresponds to the proportion of people in the standard care arm who die from other causes while having undiagnosed lung cancer.

Table S26 shows the subgroup results from the scenario analysis where it was assumed that, after the 5th year since diagnosis, people no longer died from lung cancer and had the same utilities as people with no lung cancer.

Table S26. PSA mean incremental results (screening minus standard care) from the scenario analysis where it was assumed that people no longer had lung cancer from the sixth year since diagnosis, by subgroup.

Subgroup	Life years	QALYs	Detection costs	Cost of false positives	Diagnosis and treatment costs	Total cost	ICER (total cost per QALY)
M 55-59	0.0357	0.0307	165	56	-46	175	5699
M 60-64	0.0417	0.0356	164	56	-61	159	4480
M 65-69	0.0677	0.0575	164	69	-111	122	2131
M 70-74	0.0751	0.0626	163	69	-145	88	1401
M 75-59	0.0639	0.0528	163	69	-148	84	1591
M 80	0.0395	0.0321	164	69	-103	129	4032
F 55-59	0.0386	0.0325	165	56	-46	175	5368
F 60-64	0.0458	0.0383	164	56	-63	158	4132
F 65-69	0.0744	0.0615	164	69	-114	119	1942
F 70-74	0.0795	0.0639	163	69	-153	80	1248
F 75-79	0.0680	0.0542	163	69	-162	70	1292
F 80	0.0423	0.0332	164	69	-116	117	3517

Table notes. Abbreviations: ICER: incremental cost-effectiveness ratio; QALYs: quality-adjusted life years; PSA: probabilistic sensitivity analysis.

Colour legend: light green = intervention more effective than comparator; amber = intervention more expensive than comparator; light blue = intervention cheaper than comparator.

Results are per person participating in the screening programme and refer to a lifetime horizon.

References

1. International Agency for Research on Cancer (IARC). Arsenic, Metals, Fibres, and Dusts. In *IARC Monographs on the Evaluation of Carcinogenic Risks to Humans*; International Agency for Research on Cancer (IARC): Lyon, France, 2012; Volume 100C.
2. Centers for Disease Control and Prevention. Lung Cancer Risk Factors. 2025. Available online: https://www.cdc.gov/lung-cancer/risk-factors/index.html (accessed on 25 May 2025).
3. Klebe, S.; Leigh, J.; Henderson, D.W.; Nurminen, M. Asbestos, Smoking and Lung Cancer: An Update. *Int. J. Environ. Res. Public Health* **2020**, *17*, 258. https://doi.org/10.3390/ijerph17010258.
4. Virta, R.L. Worldwide Asbestos Supply and Consumption Trends from 1900 Through 2003: U.S. Geological Survey Circular 1298. 2006. Available online: http://pubs.usgs.gov/circ/2006/1298/c1298.pdf (accessed on 26 June 2025).
5. Fazzo, L.; Binazzi, A.; Ferrante, D.; Minelli, G.; Consonni, D.; Bauleo, L.; Bruno, C.; Bugani, M.; De Santis, M.; Iavarone, I.; et al. Burden of Mortality from Asbestos-Related Diseases in Italy. *Int. J. Environ. Res. Public Health* **2021**, *18*, 10012. https://doi.org/10.3390/ijerph181910012.
6. Huh, D.-A.; Chae, W.-R.; Choi, Y.-H.; Kang, M.-S.; Lee, Y.-J.; Moon, K.-W. Disease Latency According to Asbestos Exposure Characteristics among Malignant Mesothelioma and Asbestos-Related Lung Cancer Cases in South Korea. *Int. J. Environ. Res. Public Health* **2022**, *19*, 15934. https://doi.org/10.3390/ijerph192315934.
7. Adams, S.J.; Stone, E.; Baldwin, D.R.; Vliegenthart, R.; Lee, P.; Fintelmann, F.J. Lung Cancer Screening. *Lancet* **2023**, *401*, 390–408. https://doi.org/10.1016/S0140-6736(22)01694-4.
8. Yang, W.; Qian, F.; Teng, J.; Wang, H.; Manegold, C.; Pilz, L.R.; Voigt, W.; Zhang, Y.; Ye, J.; Chen, Q.; et al. Community-Based Lung Cancer Screening with Low-Dose CT in China: Results of the Baseline Screening. *Lung Cancer* **2018**, *117*, 20–26. https://doi.org/10.1016/j.lungcan.2018.01.003.
9. Field, J.K.; Vulkan, D.; Davies, M.P.A.; Baldwin, D.R.; Brain, K.E.; Devaraj, A.; Eisen, T.; Gosney, J.; Green, B.A.; Holemans, J.A.; et al. Lung Cancer Mortality Reduction by LDCT Screening: UKLS Randomised Trial Results and International Meta-Analysis. *Lancet Reg. Health—Eur.* **2021**, *10*, 100179. https://doi.org/10.1016/j.lanepe.2021.100179.
10. Field, J.K.; Vulkan, D.; Davies, M.P.A.; Duffy, S.W.; Gabe, R. Liverpool Lung Project Lung Cancer Risk Stratification Model: Calibration and Prospective Validation. *Thorax* **2021**, *76*, 161–168. https://doi.org/10.1136/thoraxjnl-2020-215158.
11. Cassidy, A.; Myles, J.P.; van Tongeren, M.; Page, R.D.; Liloglou, T.; Duffy, S.W.; Field, J.K. The LLP Risk Model: An Individual Risk Prediction Model for Lung Cancer. *Br. J. Cancer* **2008**, *98*, 270–276. https://doi.org/10.1038/sj.bjc.6604158.
12. Barbone, F.; Barbiero, F.; Belvedere, O.; Rosolen, V.; Giangreco, M.; Zanin, T.; Pisa, F.E.; Meduri, S.; Follador, A.; Grossi, F.; et al. Impact of Low-Dose Computed Tomography Screening on Lung Cancer Mortality among Asbestos-Exposed Workers. *Int. J. Epidemiol.* **2018**, *47*, 1981–1991. https://doi.org/10.1093/ije/dyy212.
13. Veronesi, G.; Navone, N.; Novellis, P.; Dieci, E.; Toschi, L.; Velutti, L. Favorable Incremental Cost-Effectiveness Ratio for Lung Cancer Screening in Italy. *Lung Cancer* **2020**, *143*, 73–79. https://doi.org/10.1016/j.lungcan.2020.03.015.
14. Spandonaro, F.; Veronesi, G.; d'Errico, M.; Ferrara, R.; Giannarelli, D.; Graziano, P.; Polistena, B. Lung Cancer Screening Can Be a Cost Saving Public Health Measure: A Cost-Consequences Assessment for Italy. Preprint. *Social Science Research Network (SSRN)* **2024**, https://doi.org/10.2139/ssrn.5021038.
15. Behr, C.M.; Oude Wolcherink, M.J.; IJzerman, M.J.; Vliegenthart, R.; Koffijberg, H. Population-Based Screening Using Low-Dose Chest Computed Tomography: A Systematic Review of Health Economic Evaluations. *PharmacoEconomics* **2023**, *41*, 395–411. https://doi.org/10.1007/s40273-022-01238-3.
16. Fitzgerald, N.R.; Flanagan, W.M.; Evans, W.K.; Miller, A.B.; Canadian Partnership against Cancer (CPAC) Cancer Risk Management (CRM) Lung Cancer Working. Eligibility for Low-Dose Computerized Tomography

Screening among Asbestos-Exposed Individuals. *Scand. J. Work. Environ. Health* **2015**, *41*, 407–412. https://doi.org/10.5271/sjweh.3496.
17. Maisonneuve, P.; Rampinelli, C.; Bertolotti, R.; Misotti, A.; Lococo, F.; Casiraghi, M.; Spaggiari, L.; Bellomi, M.; Novellis, P.; Solinas, M.; et al. Low-Dose Computed Tomography Screening for Lung Cancer in People with Workplace Exposure to Asbestos. *Lung Cancer Amst. Neth.* **2019**, *131*, 23–30. https://doi.org/10.1016/j.lungcan.2019.03.003.
18. Markowitz, S.B. Lung Cancer Screening in Asbestos-Exposed Populations. *Int. J. Environ. Res. Public Health* **2022**, *19*, 2688. https://doi.org/10.3390/ijerph19052688.
19. Silva, M.; Picozzi, G.; Sverzellati, N.; Anglesio, S.; Bartolucci, M.; Cavigli, E.; Deliperi, A.; Falchini, M.; Falaschi, F.; Ghio, D.; et al. Low-Dose CT for Lung Cancer Screening: Position Paper from the Italian College of Thoracic Radiology. *Radiol. Med.* **2022**, *127*, 543–559. https://doi.org/10.1007/s11547-022-01471-y.
20. United States Preventive Services Task Force. Lung Cancer: Screening. 2021. Available online: https://www.uspreventiveservicestaskforce.org/uspstf/recommendation/lung-cancer-screening (accessed on 30 November 2023).
21. Regione Friuli Venezia Giulia. Registro Regionale Degli Esposti Ad Amianto [Not Publicly Available]. 2024 (accessed on 18 November 2024).
22. Fasola, G.; Belvedere, O.; Aita, M.; Zanin, T.; Follador, A.; Cassetti, P.; Meduri, S.; De Pangher, V.; Pignata, G.; Rosolen, V.; et al. Low-Dose Computed Tomography Screening for Lung Cancer and Pleural Mesothelioma in an Asbestos-Exposed Population: Baseline Results of a Prospective, Nonrandomized Feasibility Trial—An Alpe-Adria Thoracic Oncology Multidisciplinary Group Study (ATOM 002). *Oncologist* **2007**, *12*, 1215–1224. https://doi.org/10.1634/theoncologist.12-10-1215.
23. Consonni, D.; Pierobon, M.; Gail, M.; Rubagotti, M.; Rotunno, M.; Goldstein, A; Goldin, L.; Lubin, J.; Wacholder, S.; Caporaso, N.; et al. Lung Cancer Prognosis Before and After Recurrence in a Population-Based Setting. *J. Natl. Cancer Inst.* **2015**, *107*, djv059. https://doi.org/10.1093/jnci/djv059.
24. Hofer, F.; Kauczor, H.-U.; Stargardt, T. Cost-Utility Analysis of a Potential Lung Cancer Screening Program for a High-Risk Population in Germany: A Modelling Approach. *Lung Cancer* **2018**, *124*, 189–198. https://doi.org/10.1016/j.lungcan.2018.07.036.
25. Regione Friuli Venezia Giulia. Registro Tumori FVG. 2026. https://www.cro.sanita.fvg.it/it/ricercatori/registro_tumori.html (accessed on 28 January 2026)
26. Associazione Italiana di Oncologia Medica (AIOM); Associazione Italiana Registri Tumori (AIRTUM); PASSI; SIAPEC-IAP. I Numeri Del Cancro in Italia 2020. Available online: https://www.epicentro.iss.it/tumori/pdf/2020_Numeri_Cancro-operatori-web.pdf (accessed on 17 June 2024).
27. Meregaglia, M.; Malandrini, F.; Finch, A.P.; Ciani, O.; Jommi, C. EQ-5D-5L Population Norms for Italy. *Appl. Health Econ. Health Policy* **2023**, *21*, 289–303. https://doi.org/10.1007/s40258-022-00772-7.
28. Maheswaran, H.; Petrou, S.; Rees, K.; Stranges, S. Estimating EQ-5D Utility Values for Major Health Behavioural Risk Factors in England. *J. Epidemiol. Community Health* **2013**, *67*, 172–180. https://doi.org/10.1136/jech-2012-201019.
29. Vogl, M.; Wenig, C.M.; Leidl, R.; Pokhrel, S. Smoking and Health-Related Quality of Life in English General Population: Implications for Economic Evaluations. *BMC Public Health* **2012**, *12*, 203. https://doi.org/10.1186/1471-2458-12-203.
30. Casadei, G.; Tolley, K.; Bettio, M.; Bozza, F.; Cafaro, A.; Dall'Ara, M.C.; Pedrazzini, A.; Scintu, V.; Zanotti, G.; Bignamini, A.A. Investigation of Health-Related Quality of Life Outcomes in Cancer Patients: Findings from an Observational Study Using the EQ-5D in Italy. *SN Compr. Clin. Med.* **2020**, *2*, 1579–1584. https://doi.org/10.1007/s42399-020-00449-z.
31. Tramontano, A.C.; Schrag, D.L.; Malin, J.K.; Miller, M.C.; Weeks, J.C.; Swan, J.S.; McMahon, P.M. Catalog and Comparison of Societal Preferences (Utilities) for Lung Cancer Health States: Results from the Cancer Care

Outcomes Research and Surveillance (CanCORS) Study. *Med. Decis. Mak.* **2015**, *35*, 371–387. https://doi.org/10.1177/0272989X15570364.
32. Snowsill, T.; Yang, H.; Griffin, E.; Long, L.; Varley-Campbell, J.; Coelho, H.; Robinson, S.; Hyde, C. Low-Dose Computed Tomography for Lung Cancer Screening in High-Risk Populations: A Systematic Review and Economic Evaluation. *Health Technol. Assess.* **2018**, *22*, 1–276. https://doi.org/10.3310/hta22690.
33. Regione Friuli Venezia Giulia. Nomenclatore Tariffario Della Specialistica Ambulatoriale Della Regione Friuli Venezia Giulia. 2019. Available online: https://www.regione.fvg.it/rafvg/export/sites/default/RAFVG/salute-sociale/sistema-sociale-sanitario/FOGLIA51/allegati/28112019_nomenclatore_FVG_2019_in_vigore.pdf (accessed on 22 March 2024).
34. Regione Friuli Venezia Giulia. Tariffario Regionale per Le Prestazioni Di Assistenza Ospedaliera. 2019. Available online: https://www.regione.fvg.it/rafvg/export/sites/default/RAFVG/salute-sociale/sistema-sociale-sanitario/FOGLIA50/allegati/27122019_Tariffario_regionale_prestazioni_assistenza_ospedaliera.pdf (accessed on 22 March 2024).
35. European Central Bank. HICP—Hospital Services, Italy, Annual. 2024. Available online: https://data.ecb.europa.eu/data/concepts/hospital-services?searchTerm=&sort=relevance&pageSize=10&filterType=basic&showDatasetModal=false&filtersReset=false&resetAll=false&tags_array%5B%5D=Hospital%20services&reference_area_name%5B%5D=Italy&frequency%5B%5D=A (accessed on 10 February 2024).
36. European Central Bank. HICP—Out-Patient Services, Italy, Annual. 2024. Available online: https://data.ecb.europa.eu/data/concepts/out-patient-services?searchTerm=&sort=relevance&pageSize=10&filterType=basic&showDatasetModal=false&filtersReset=false&resetAll=false&tags_array%5B%5D=Out-patient%20services&reference_area_name%5B%5D=Italy&frequency%5B%5D=A (accessed on 10 February 2024).
37. International Agency for Research on Cancer. Benefits and Harms of Lung Cancer Screening. 2019. Available online: https://www.iarc.who.int/wp-content/uploads/2019/04/IARC-Benefits-and-Harms-of-Lung-Cancer-Screening.pdf (accessed on 11 January 2025).
38. Pinsky, P.F.; Gierada, D.S.; Hocking, W.; Patz, E.F., Jr.; Kramer, B.S. National Lung Screening Trial Findings by Age: Medicare-Eligible Versus Under-65 Population. *Ann. Intern. Med.* **2014**, *161*, 627–633. https://doi.org/10.7326/M14-1484.
39. National Lung Screening Trial Research Team; Aberle, D.R.; Adams, A.M.; Berg, C.D.; Black, W.C.; Clapp, J.D.; Fagerstrom, R.M.; Gareen, I.F.; Gatsonis, C.; Marcus, P.M.; et al. Reduced Lung-Cancer Mortality with Low-Dose Computed Tomographic Screening. *N. Engl. J. Med.* **2011**, *365*, 395–409. https://doi.org/10.1056/NEJMoa1102873.
40. Croswell, J.M.; Baker, S.G.; Marcus, P.M.; Clapp, J.D.; Kramer, B.S. Cumulative Incidence of False-Positive Test Results in Lung Cancer Screening. *Ann. Intern. Med.* **2010**, *152*, 505–512. https://doi.org/10.7326/0003-4819-152-8-201004200-00007.
41. Azienda Sanitaria Universitaria Giuliano Isontina (ASUGI). PDTA per Le Neoplasie Polmonari. 2024. Available online: https://asugi.sanita.fvg.it/export/sites/aas1/it/documenti/all_pdta/PDTA_Neoplasie_polmonari_2025.pdf (accessed on 25 September 2025).
42. American College of Radiology. Lung-RADS Version 1.1. Available online: https://www.acr.org/Clinical-Resources/Clinical-Tools-and-Reference/Reporting-and-Data-Systems/Lung-RADS (accessed on 20 November 2023).

43. Russo, P.; Zanuzzi, M.; Carletto, A.; Sammarco, A.; Romano, F.; Manca, A. Role of Economic Evaluations on Pricing of Medicines Reimbursed by the Italian National Health Service. *Pharmacoeconomics* **2023**, *41*, 107–117. https://doi.org/10.1007/s40273-022-01215-w.
44. Fattore, G. Proposta di linee guida per la valutazione economica degli interventi sanitari in Italia. *PharmacoEconomics Ital. Res. Artic.* **2009**, *11*, 83–93. https://doi.org/10.1007/BF03320660.
45. Agenzia Italiana del Farmaco (AIFA). Linee Guida per La Compilazione Del Dossier a Supporto Della Domanda Di Rimborsabilità e Prezzo Di Un Medicinale. Versione 1.0. 2020. Available online: https://www.aifa.gov.it/documents/20142/1307543/2021.01.22_estratto_linee_guida_sezione_E.pdf (accessed on 30 November 2023).
46. *Microsoft Excel*, Microsoft 365 (Version 2602); Microsoft Corporation: Redmond, WA, USA, 2024.
47. Infante, M.; Lutman, F.R.; Cavuto, S.; Brambilla, G.; Chiesa, G.; Passera, E.; Angeli, E.; Chiarenza, M.; Aranzulla, G.; Cariboni, U.; et al. Lung Cancer Screening with Spiral CT: Baseline Results of the Randomized DANTE Trial. *Lung Cancer Amst. Neth.* **2008**, *59*, 355–363. https://doi.org/10.1016/j.lungcan.2007.08.040.
48. Falk Hvidberg, M.; Hernández Alava, M. Catalogues of EQ-5D-3L Health-Related Quality of Life Scores for 199 Chronic Conditions and Health Risks for Use in the UK and the USA. *PharmacoEconomics* **2023**, *41*, 1287–1388. https://doi.org/10.1007/s40273-023-01285-4.
49. Hammer, M.M.; Byrne, S.C.; Kong, C.Y. Factors Influencing the False Positive Rate in CT Lung Cancer Screening. *Acad. Radiol.* **2022**, *29*, S18–S22. https://doi.org/10.1016/j.acra.2020.07.040.
50. Buja, A.; Rivera, M.; De Polo, A.; di Brino, E.; Marchetti, M.; Scioni, M.; Pasello, G.; Bortolami, A.; Rebba, V.; Schiavon, M.; et al. Estimated Direct Costs of Non-Small Cell Lung Cancer by Stage at Diagnosis and Disease Management Phase: A Whole-Disease Model. *Thorac. Cancer* **2021**, *12*, 13–20. https://doi.org/10.1111/1759-7714.13616.
51. Sun, L.; Peng, X.; Li, S.; Huang, Z. Cost-Effectiveness Thresholds or Decision-Making Threshold: A Novel Perspective. *Cost Eff. Resour. Alloc.* **2023**, *21*, 72. https://doi.org/10.1186/s12962-023-00472-6.
52. Barlow, W.E. Overview of Methods to Estimate the Medical Costs of Cancer. *Med. Care* **2009**, *47*, S33–S36. https://doi.org/10.1097/MLR.0b013e3181a2d847.
53. Hwang, J.-S.; Hu, T.-H.; Lee, L.J.-H.; Wang, J.-D. Estimating Lifetime Medical Costs from Censored Claims Data. *Health Econ.* **2017**, *26*, e332–e344. https://doi.org/10.1002/hec.3512.
54. Wu, T.-Y.; Chung, C.-H.; Lin, C.-N.; Hwang, J.-S.; Wang, J.-D. Lifetime Risks, Loss of Life Expectancy, and Health Care Expenditures for 19 Types of Cancer in Taiwan. *Clin. Epidemiol.* **2018**, *10*, 581–591. https://doi.org/10.2147/CLEP.S155601.
55. Park, H.-Y.; Hwang, J.; Kim, D.-H.; Jeon, S.M.; Choi, S.H.; Kwon, J.-W. Lifetime Survival and Medical Costs of Lung Cancer: A Semi-Parametric Estimation from South Korea. *BMC Cancer* **2020**, *20*, 846. https://doi.org/10.1186/s12885-020-07353-8.
56. Godoy, M.C.B.; Pereira, H.A.C.; Carter, B.W.; Wu, C.C.; Erasmus, J.J. Incidental Findings in Lung Cancer Screening: Which Ones Are Relevant? *Semin. Roentgenol.* **2017**, *52*, 156–160. https://doi.org/10.1053/j.ro.2017.06.007.
57. Hewitt, R.J.; Bartlett, E.C.; Ganatra, R.; Butt, H.; Kouranos, V.; Chua, F.; Kokosi, M.; Molyneaux, P.L.; Desai, S.R.; Wells, A.U.; et al. Lung Cancer Screening Provides an Opportunity for Early Diagnosis and Treatment of Interstitial Lung Disease. *Thorax* **2022**, *77*, 1149–1151. https://doi.org/10.1136/thorax-2022-219068.
58. Peters, J.L.; Snowsill, T.M.; Griffin, E.; Robinson, S.; Hyde, C.J. Variation in Model-Based Economic Evaluations of Low-Dose Computed Tomography Screening for Lung Cancer: A Methodological Review. *Value Health* **2022**, *25*, 656–665. https://doi.org/10.1016/j.jval.2021.11.1352.
59. Thomas, C.; Heathcote, L.; Sun, Y.; Callister, M.E.J.; Kitt, J.; Rossi, S.H.; Shinkins, B.; Usher-Smith, J.A.; Whyte, S.; Stewart, G.D. Cost-Effectiveness of One-off Upper Abdominal CT Screening as an Add-on to Lung Cancer Screening in England. *Br. J. Cancer* **2025**, *133*, 239–247. https://doi.org/10.1038/s41416-025-03043-z.

60. Exeter Test Group and Health Economics Group. *Interim Report on the Cost-Effectiveness of Low Dose Computed Tomography (LDCT) Screening for Lung Cancer in High Risk Individuals*; Version 1.3; The UK National Screening Committee: London, UK, 2022. Available online: https://view-health-screening-recommendations.service.gov.uk/document/2729a951-ad3b-473f-8285-3b9e30786612/download (accessed on 20 September 2025).
61. Evans, W.K.; Wolfson, M.C.; Flanagan, W.M.; Shin, J.; Goffin, J.; Miller, A.B.; Asakawa, K.; Earle, C.; Mittmann, N.; Fairclough, L.; et al. Canadian Cancer Risk Management Model: Evaluation of Cancer Control. *Int. J. Technol. Assess. Health Care* **2013**, *29*, 131–139. https://doi.org/10.1017/S0266462313000044.
62. Cellina, M.; Cacioppa, L.M.; Cè, M.; Chiarpenello, V.; Costa, M.; Vincenzo, Z.; Pais, D.; Bausano, M.V.; Rossini, N.; Bruno, A.; et al. Artificial Intelligence in Lung Cancer Screening: The Future Is Now. *Cancers* **2023**, *15*, 4344. https://doi.org/10.3390/cancers15174344.
63. Brouwer, W.; van Baal, P. Moving Forward with Taking a Societal Perspective: A Themed Issue on Productivity Costs, Consumption Costs and Informal Care Costs. *PharmacoEconomics* **2023**, *41*, 1027–1030. https://doi.org/10.1007/s40273-023-01307-1.
64. Sittimart, M.; Rattanavipapong, W.; Mirelman, A.J.; Hung, T.M.; Dabak, S.; Downey, L.E.; Jit, M.; Teerawattananon, Y.; Turner, H.C. An Overview of the Perspectives Used in Health Economic Evaluations. *Cost Eff. Resour. Alloc. CE* **2024**, *22*, 41. https://doi.org/10.1186/s12962-024-00552-1.
65. Longo, C.J. Societal Perspectives and Real-World Cost-Effectiveness: Expanding the Scope of Health Economics Inquiry. *Curr. Oncol.* **2022**, *30*, 233–235. https://doi.org/10.3390/curroncol30010018.
66. Krol, M.; Brouwer, W.; Rutten, F. Productivity Costs in Economic Evaluations: Past, Present, Future. *PharmacoEconomics* **2013**, *31*, 537–549. https://doi.org/10.1007/s40273-013-0056-3.
67. Avanceña, A.L.V.; Prosser, L.A. Innovations in Cost-Effectiveness Analysis That Advance Equity Can Expand Its Use in Health Policy. *BMJ Glob. Health* **2022**, *7*, e008140. https://doi.org/10.1136/bmjgh-2021-008140.
68. Regione Friuli Venezia Giulia. Registro Tumori FVG. 2024. https://www.cro.sanita.fvg.it/it/ricercatori/registro_tumori.html (accessed on 28 March 2024)
69. ISTAT. Popolazione Residente per Sesso, Età e Stato Civile al 1° Gennaio 2019. Available online: https://demo.istat.it/app/?l=it&a=2019&i=POS (accessed on 13 February 2026).
70. ISTAT. Tavole Di Mortalità: Singole Età. Friuli Venezia Giulia 2022, Maschi e Femmine. 2023. Available online: http://dati.istat.it/Index.aspx?DataSetCode=DCIS_MORTALITA1# (accessed on 17 February 2024).
71. ISTAT. Cause Di Morte in Italia: Anno 2021. 2024. Available online: https://www.istat.it/it/files//2024/06/Report-cause-di-morte-Anno-2021.pdf (accessed on 5 December 2024).
72. Serraino, D.; Bidoli, E.; Dal Maso, L.; Toffolutti, F.; Angelin, T.; De Santis, E.; Forgiarini, O. I Tumori in Friuli Venezia Giulia: 25 Anni Di Registrazione, 1998–2023. 2023. Available online: https://www.cro.sanita.fvg.it/export/sites/cro/it/ricercatori/documenti/registro_tumori/registro-tumori-fvg-25-anni.pdf (accessed on 24 June 2024).
73. Ferrante, D.; Angelini, A.; Barbiero, F.; Barbone, F.; Bauleo, L.; Binazzi, A.; Bovenzi, M.; Bruno, C.; Casotto, V.; Cernigliaro, A.; et al. Cause Specific Mortality in an Italian Pool of Asbestos Workers Cohorts. *Am. J. Ind. Med.* **2024**, *67*, 31–43. https://doi.org/10.1002/ajim.23546.
74. ISTAT. Popolazione Residente al 1° Gennaio. Friuli Venezia Giulia, 2023. Available online: http://dati.istat.it/Index.aspx?DataSetCode=DCIS_POPRES1# (accessed on 16 February 2024).
75. van den Bergh, K.; Essink-Bot, M.L.; Borsboom, G.J.J.M.; Th Scholten, E.; Prokop, M.; de Koning, H.J.; van Klaveren, R.J. Short-Term Health-Related Quality of Life Consequences in a Lung Cancer CT Screening Trial (NELSON). *Br. J. Cancer* **2010**, *102*, 27–34. https://doi.org/10.1038/sj.bjc.6605459.
76. Mazzone, P.J.; Obuchowski, N.; Fu, A.Z.; Phillips, M.; Meziane, M. Quality of Life and Healthcare Use in a Randomized Controlled Lung Cancer Screening Study. *Ann. Am. Thorac. Soc.* **2013**, *10*, 324–329. https://doi.org/10.1513/AnnalsATS.201301-007OC.

77. Field, J.K.; Duffy, S.W.; Baldwin, D.R.; Brain, K.E.; Devaraj, A.; Eisen, T.; Green, B.A.; Holemans, J.A.; Kavanagh, T.; Kerr, K.M.; et al. The UK Lung Cancer Screening Trial: A Pilot Randomised Controlled Trial of Low-Dose Computed Tomography Screening for the Early Detection of Lung Cancer. *Health Technol. Assess.* **2016**, *20*, 1–146. https://doi.org/10.3310/hta20400.
78. O'Keeffe, L.; Taylor, G.; Huxley, R.; Mitchell, P.; Woodward, M.; Peters, S. Smoking as a Risk Factor for Lung Cancer in Women and Men: A Systematic Review and Meta-Analysis. *BMJ Open* **2018**, *8*, e021611. https://doi.org/10.1136/bmjopen-2018-021611.
79. Olsson, A.C.; Vermeulen, R.; Schütz, J.; Kromhout, H.; Pesch, B.; Peters, S.; Behrens, T.; Portengen, L.; Mirabelli, D.; Gustavsson, P.; et al. Exposure-Response Analyses of Asbestos and Lung Cancer Subtypes in a Pooled Analysis of Case-Control Studies. *Epidemiology* **2017**, *28*, 288–299. https://doi.org/10.1097/EDE.0000000000000604.
80. Lopes Pegna, A.; Picozzi, G.; Mascalchi, M.; Maria Carozzi, F.; Carrozzi, L.; Comin, C.; Spinelli, C.; Falaschi, F.; Grazzini, M.; Innocenti, F.; et al. Design, Recruitment and Baseline Results of the ITALUNG Trial for Lung Cancer Screening with Low-Dose CT. *Lung Cancer Amst. Neth.* **2009**, *64*, 34–40. https://doi.org/10.1016/j.lungcan.2008.07.003.
81. Paci, E.; Puliti, D.; Pegna, A.L.; Carrozzi, L.; Picozzi, G.; Falaschi, F.; Pistelli, F.; Aquilini, F.; Ocello, C.; Zappa, M.; et al. Mortality, Survival and Incidence Rates in the ITALUNG Randomised Lung Cancer Screening Trial. *Thorax* **2017**, *72*, 825–831. https://doi.org/10.1136/thoraxjnl-2016-209825.
82. Gidwani, R.; Russell, L.B. Estimating Transition Probabilities from Published Evidence: A Tutorial for Decision Modelers. *PharmacoEconomics* **2020**, *38*, 1153–1164. https://doi.org/10.1007/s40273-020-00937-z.
